# Pak Choi (*Brassica rapa* L. subsp. *chinensis*): Phytochemical Diversity, Biological Activities, and Emerging Applications in Nutrition, Health and Cosmetics

**DOI:** 10.3390/molecules31213919

**Published:** 2026-10-30

**Authors:** Magdalena Kniaziewicz, Aleksandra Łukaszyk, Anita Kanik, Małgorzata Tatarczak-Michalewska, Inga Kwiecień, Katarzyna Czarnek, Agnieszka Szopa, Eliza Blicharska

**Affiliations:** 1 Department of Pathobiochemistry and Interdisciplinary Applications of Ion Chromatography, Medical University of Lublin, 1 Chodźki St., 20-093 Lublin, Poland; magdalena.kniaziewicz@gmail.com (M.K.); malgorzata.tatarczak-michalewska@umlub.pl (M.T.-M.); 2 Doctoral School of the Medical University of Lublin, 7 Witolda Chodźki St., 20-093 Lublin, Poland; 3 Department of Medicinal Plant and Mushroom Biotechnology, Faculty of Pharmacy, Jagiellonian University Medical College, 9 Medyczna St., 30-688 Cracow, Poland; aleksandra.lukaszyk@student.uj.edu.pl (A.Ł.); aanitakanik@gmail.com (A.K.); inga.kwiecien@uj.edu.pl (I.K.); 4 Doctoral School of Medical and Health Sciences, Jagiellonian University Medical College, 16 Św. Łazarza St., 30-530 Cracow, Poland; 5 Institute of Medical Science, Faculty of Medical Sciences, The John Paul II Catholic University of Lublin, 1H Konstantynów St., 20-708 Lublin, Poland; katarzyna.czarnek@kul.pl

**Keywords:** pak choi, *Brassica rapa* subsp. *chinensis*, glucosinolates, sulforaphane, phytochemistry, functional food, nutraceuticals, plant biotechnology, cosmetics

## Abstract

*Brassica rapa* L. subsp. *chinensis* (pak choi) is an important leafy vegetable with increasing global relevance as a source of biologically active phytochemicals. Despite extensive research on individual compounds, a comprehensive and integrative understanding of its phytochemical profile and functional potential remains limited. This review critically synthesizes current knowledge on the chemical composition, biological activities, and emerging applications of pak choi, with particular emphasis on its nutraceutical and biotechnological significance. Pak choi is characterized by a diverse spectrum of bioactive constituents, including glucosinolates, phenolic compounds, carotenoids, vitamins, minerals, and amino acids, which collectively contribute to its antioxidant, anti-inflammatory, chemopreventive, and metabolic regulatory effects. A major challenge highlighted in this analysis is the high variability of phytochemical composition, driven by genotype, developmental stage, environmental conditions, cultivation systems, and post-harvest processing. Importantly, this review advances current understanding by integrating phytochemical, biological and technological perspectives, emphasizing the interplay between compound composition, bioactivity, and processing-dependent transformations. Particular attention is given to glucosinolate-derived metabolites and their role in modulating key molecular pathways. Furthermore, both beneficial and adverse aspects of pak choi consumption are addressed, including antinutritional factors, contamination risks, and the impact of culinary processing on compound stability and bioavailability. Emerging applications in functional foods, nutraceuticals, cosmetics and plant biotechnology are also discussed, including the potential of in vitro culture systems and targeted metabolite production. Taken together, pak choi represents a multifunctional plant resource at the interface of food science, pharmacology, and biotechnology, with strong potential for future translational applications.

## 1. Introduction

Plant systematics enables the classification of plant species into groups based on shared characteristics, facilitating the understanding of plant diversity. The foundations of modern taxonomy were established by Joseph Pitton de Tournefort and later formalized by Carl Linnaeus in 1753. Over time, biological classification systems evolved, leading to the recognition of the plant family traditionally known as Cruciferae, currently referred to as *Brassicaceae* [1].

The *Brassicaceae* family comprises approximately 350 genera and nearly 4000 species, making it one of the most economically and nutritionally significant plant groups worldwide [2,3]. Several relevant examples of somatic hybridization have been reported within this family [4]. Genetic diversity within the *Brassicaceae* family is reflected in morphological, biochemical, and evolutionary traits. The genus *Brassica* includes key crop species such as *Brassica napus* L., *Brassica oleracea* L., and *Brassica rapa* L., which are widely utilized as nutritional resources worldwide [5]. Hybridization events between species have led to the formation of amphidiploids, contributing to the diversity observed within this group.

Cytogenetic studies have established the genome composition and ploidy levels of major *Brassicaceae* crops (Table 1). *Brassica nigra* (L.) Koch is characterized by the BB genome (2*n* = 16), *Brassica oleracea* L. by the CC genome (2*n* = 18), and *Brassica rapa* L. (formerly *B. campestris*) by the AA genome (2*n* = 20) (Table 1). The latter species comprises numerous cultivated forms, including *Brassica rapa* L. subsp. *chinensis* (syn. var. *chinensis*), commonly known as pak choi, which is extensively cultivated in Asia [1,5].

Pak choi (*Brassica rapa* L. subsp. *chinensis*) is a leafy vegetable forming a rosette of edible aboveground parts, typically harvested at the vegetative stage before flowering [6]. The plant is characterized by thickened petioles and tender leaf blades, exhibiting green or white coloration depending on the cultivar and growing conditions [7].

This species is known by various common names, including pak choi, pak-choi, bok choy [8,9], and Chinese cabbage [10], which may lead to confusion with other *Brassica* vegetables. Nomenclature also varies regionally; for example, in Thailand it is referred to as Pak Keang Tung [11].

Pak choi is widely consumed in Asia, particularly in China, Korea, Japan, Vietnam, Malaysia, and Indonesia [5,6,8], where it constitutes a staple component of the daily diet. Its cultivation has also expanded to Africa (e.g., Kenya, Tanzania, Nigeria) and Western countries, including Europe and North America, reflecting its increasing global popularity [10,12,13]. The vegetable is typically consumed after thermal processing (e.g., stir-frying, blanching, boiling), whereas raw consumption is less common [6].

The growing global demand for *Brassicaceae* vegetables highlights their economic importance as sources of food and agricultural products [1,3,5]. Pak choi is particularly valued due to its short cultivation cycle, adaptability to diverse environmental conditions, and consumer acceptance, contributing to its increasing production worldwide [10,14].

Pak choi has played a significant role in Asian agriculture and nutrition for over a millennium, particularly in coastal regions [8]. It is currently one of the most widely cultivated cruciferous vegetables, accounting for approximately 30–40% of vegetable production areas in China. Cultivation is concentrated in different climatic zones, with green-petiole cultivars predominating in northern regions and white-petiole forms in southeastern areas [7]. In addition to open-field production, *B. rapa* subsp. *chinensis* is commonly cultivated in controlled environments, including greenhouse systems, enabling year-round availability [7,15]. Although traditionally consumed in Asia, its cultivation and consumption have increased globally, particularly in Europe over the past centuries [9].

The growing interest in pak choi is driven not only by its agronomic advantages but also by its rich nutritional profile and associated health benefits. However, considerable phenotypic variability and differences in chemical composition among cultivars highlight the need for comprehensive and systematic investigations of its dietary and therapeutic potential [16].

Unlike previous reviews that have predominantly addressed *Brassica* vegetables as a group or focused on individual phytochemical classes and selected biological effects, the present review provides an integrated assessment specifically centered on *B. rapa* subsp. *chinensis*. By linking phytochemical diversity with biological activity, processing-dependent transformations, safety considerations, and emerging nutraceutical, cosmetic, and biotechnological applications, this review identifies important gaps between compositional research and evidence supporting health-related applications. Particular emphasis is placed on cultivar-dependent variability, bioavailability, the level of experimental evidence supporting biological effects, and opportunities for biotechnology-assisted enhancement of valuable phytochemicals.

## 2. Literature Search Methodology

This review was based on a comprehensive survey of the available scientific literature concerning *Brassica rapa* L. subsp. *chinensis* (pak choi). No strict limitations regarding publication date were imposed; however, particular emphasis was placed on studies published between 2016 and 2026, reflecting the most recent advances in phytochemistry, biological activity, nutrition, food processing, and biotechnology. Earlier publications were also included when they provided fundamental taxonomic, biochemical, or biotechnological information that has served as the basis for subsequent research.

The literature search was conducted using major scientific databases, including PubMed, Scopus, ScienceDirect, SpringerLink, Taylor & Francis Online, Wiley Online Library, Oxford Academic, Frontiers, and Google Scholar. Searches were performed using combinations of keywords such as “pak choi”, “bok choy”, “*Brassica chinensis*”, “*Brassica rapa* subsp. *chinensis*”, “glucosinolate”, “isothiocyanate”, “sulforaphane”, “polyphenols”, “antioxidant activity”, “nutrition”, “functional food”, “microgreens”, “plant biotechnology”, and “cosmetics”. Additional relevant publications were identified through manual screening of reference lists from primary research articles and review papers.

The review was based predominantly on English-language publications; however, selected studies published in other languages were also considered when relevant information was available and could be reliably translated.

As this work is a narrative review rather than a systematic review, the PRISMA protocol was not applied. Nevertheless, the literature was critically assessed with particular attention to scientific relevance, methodological quality, and consistency of reported findings. Publications in which *Brassica rapa* subsp. *pekinensis* (Chinese cabbage) was incorrectly identified as pak choi were excluded to ensure taxonomic accuracy and consistency throughout the review.

Particular attention was devoted to studies reporting phytochemical composition, biological activities, health-related effects, processing-induced changes, biotechnological applications, and emerging nutraceutical and cosmetic uses of pak choi.

## 3. Importance of Cruciferous Vegetables in Human Nutrition

Socio-cultural nutritional habits are shaped by socio-cultural, economic, and regional factors, which strongly influence the consumption of plant-based foods and consequently health outcomes [17]. The widespread adoption of Western dietary habits, characterized by a high intake of sugars, saturated fats, and processed foods, has been associated with the increasing prevalence of obesity, non-alcoholic fatty liver disease, oxidative stress, and chronic inflammation [18,19]. Insufficient consumption of fruits and vegetables remains a major global health concern, contributing significantly to disease burden and mortality worldwide [20].

In response, dietary recommendations from organizations such as WHO and FAO emphasize the regular consumption of fresh, minimally processed plant foods, particularly vegetables, as an essential strategy for preventing metabolic disorders and maintaining overall health [20,21]. Vegetables are valuable not only as sources of primary metabolites but also due to their high content of secondary bioactive compounds, including polyphenols, glucosinolates, carotenoids, vitamins, and minerals, which exhibit antioxidant and anti-inflammatory properties [3,16].

Cruciferous vegetables, especially those belonging to the *Brassicaceae* family, occupy an important position in human nutrition due to their characteristic phytochemical profile. In Asian dietary patterns, these vegetables constitute a fundamental component of daily food intake, with reported consumption levels reaching approximately 320 g of vegetables per person per day in China, including a substantial proportion of cruciferous species such as pak choi [22,23]. Their popularity results from a combination of accessibility, culinary versatility, short preparation time, and well-documented health benefits.

Among these, *B. rapa* subsp. *chinensis* occupies a prominent position, being popularly consumed in Asian cuisine and increasingly incorporated into dietary patterns in other regions [7,8,24]. Its versatility enables its use in a variety of culinary forms, including fermented products such as kimchi, which additionally contribute to gut microbiota modulation and metabolic regulation [25,26].

Beyond its nutritional role, pak choi has attracted scientific interest due to its diverse biological effects, including antioxidant, anti-inflammatory, and metabolic regulatory activities, largely attributed to its rich content of flavonoids, glucosinolates, pigments, and micronutrients [27,28]. These properties highlight its potential as a functional food in the prevention of chronic diseases.

Consequently, contemporary research increasingly shifts focus from macronutrient composition toward the role of phytochemicals and secondary metabolites, positioning *Brassicaceae* vegetables, including pak choi, as important components of modern, health-oriented dietary strategies [16].

The aim of this review is to comprehensively collect, critically evaluate, and systematize current knowledge on *B. rapa* subsp. *chinensis*, with a particular focus on its phytochemical profile, nutritional properties, and emerging applications in human health and nutrition. Unlike previous studies that primarily concentrate on selected aspects such as metabolomic diversity or individual groups of bioactive compounds (e.g., glucosinolates or flavonoids), this review integrates multidisciplinary evidence spanning plant chemistry, dietary relevance, cultivation-dependent variability, and preventive health potential [5,7,9,16,19,20,23,26,28].

Pak choi is increasingly recognized not only as a dietary vegetable but also as a rich source of bioactive phytochemicals, including glucosinolates, phenolic compounds, vitamins, and minerals, which have been associated with antioxidant, anti-inflammatory, and chemoprotective effects [1,29]. These compounds play a key role in the prevention of chronic diseases, including cardiovascular, metabolic, and neurodegenerative disorders, as demonstrated for cruciferous vegetables in general [30].

Although a growing number of studies have investigated the metabolic composition of pak choi using advanced analytical approaches, including metabolomics and transcriptomics [16,31,32], the available literature remains fragmented and often limited to specific cultivars, developmental stages, or individual classes of compounds. Moreover, existing studies highlight substantial variability in phytochemical content among cultivars, including differences in glucosinolate profiles and phenolic composition, which significantly influence their biological activity and nutritional value [33,34].

In this context, the present review addresses the need for an integrated and critical synthesis of current knowledge by combining data on chemical composition, biological activity, dietary relevance, and agronomic factors affecting metabolite accumulation. Particular attention is given to linking phytochemical diversity with potential health outcomes, as well as to identifying knowledge gaps related to standardization, bioavailability, and the mechanistic basis of biological effects.

Furthermore, this work highlights emerging directions for the application of pak choi as a functional food and nutraceutical resource, thereby extending its relevance beyond traditional dietary use. By providing a comprehensive and interdisciplinary perspective, this review aims to support future research, facilitate the development of high-value cultivars, and promote the utilization of pak choi in modern health-oriented nutrition strategies.

## 4. Taxonomy, Botanical and Geographical Data

*B. rapa* var. *chinensis* is an annual or biennial variety of the species *Brassica rapa* L. Initially, it was a separate species called *Brassica chinensis* L. However, it was later reclassified as a variety of *Brassica rapa* L. in the “Flora of China” [35]. *Brassica rapa* ssp. *chinensis* is also a synonym for *Brassica campestris* ssp. *chinensis* [36]. This plant is often called bok choy, pak choi, celery mustard, Chinese mustard, chongee, and pe-tsai [37]. This plant is termed by numerous synonyms, including the compound denomination “pakchoi”, “pak-choi” with a dash between the terms or simple pak choi [8,9], and Chinese cabbage [10]. Nomenclature may vary across different geographic regions. In Thailand, the wide spread name is Pak Keang Tung [11]. Unfortunately, the nomenclature of this vegetable is often confused with that of other cabbage varieties, which may lead to misinterpretations and incorrect conclusions regarding the properties of this particular subspecies. In this study, scientific literature from various authors was carefully analyzed to identify and clarify such inconsistencies.

**Figure 1:**
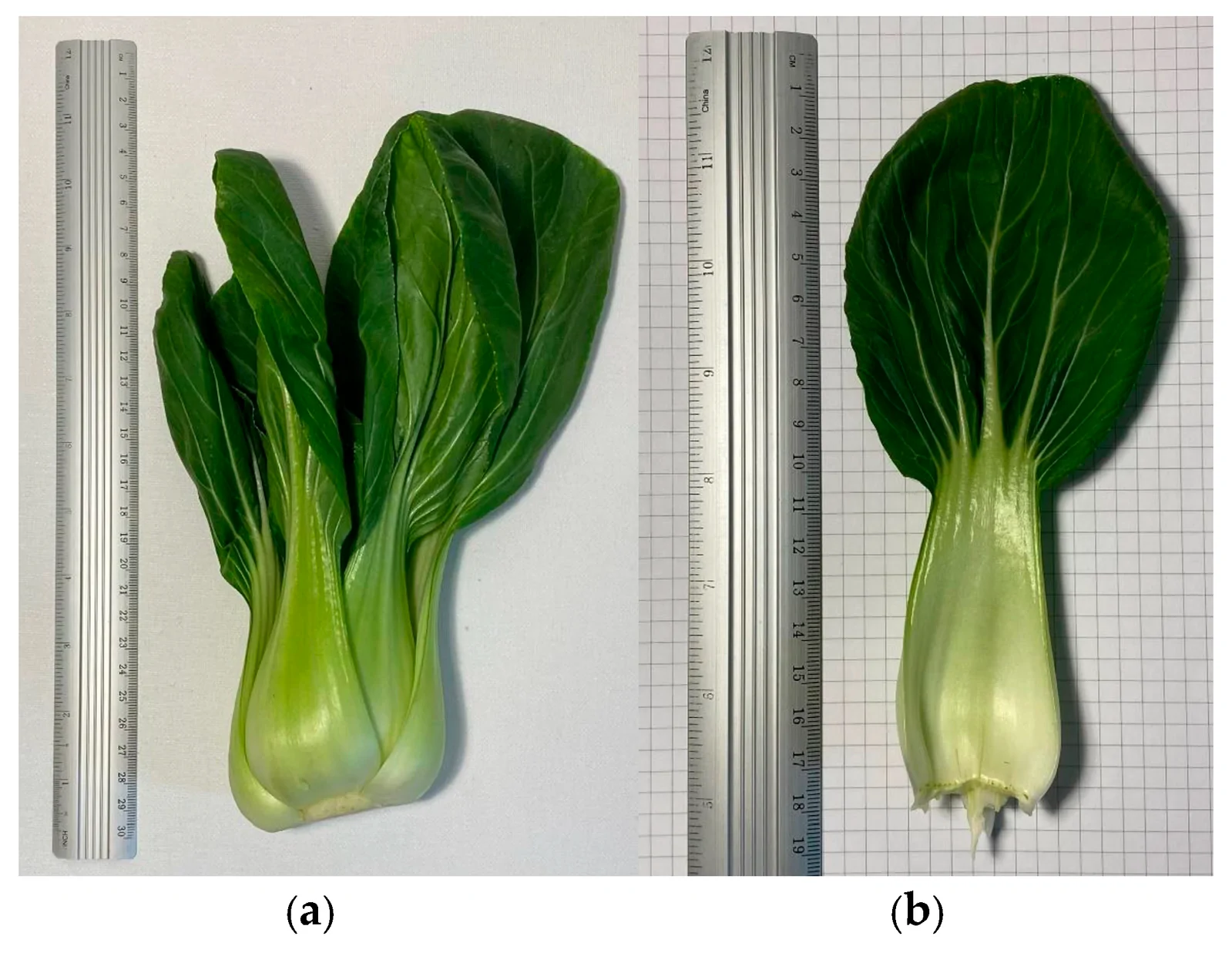
*Brassica rapa* var. *chinensis*—(**a**) morphological appearance of the plant and (**b**) leaf (photo by Anita Kanik).

Many pak choi cultivars are collected in plant gene banks due to differences in morphological characteristics. Pak choi has leaves that do not form a head, distinguishing it from Chinese cabbage [34]. The ideal pak choi is characterized by a dark green, entire, rounded leaf blade and a white, fleshy petiole. It has a cylindrical main root and usually more than 20 basal leaves that form a rosette (Figure 1) [37]. There are also dark purple and red cultivated varieties [38]. Pak choi has many cultivars that differ in the appearance and color of the leaves and petiole (Table 2) [39].

Based on data from the Federal Research Center VIR (Vavilov All-Russian Institute of Plant Genetic Resources; St. Petersburg, Russia), which maintains a rich collection of genetic resources of cultivated plants and their wild relatives, the following pak choi cultivars can be distinguished: Syusman, Yutsai, Taisai, Piorbai, Leychoi, and Joychoi (Table 3). The most typical pak choi cultivar is Syusman. It is characterized by a compact leaf rosette, a thick, relatively short (from 8 to 13 cm) and very wide (from 5 to 7 cm), light green leaf petiole. The Yutsai type is a relatively late-maturing cultivar. The Taisai cultivar has a closed leaf rosette with a long (over 25 cm), pale green or white leaf petiole. The Piorbai type is characterized by a semi-spreading leaf rosette with more numerous, dark green leaves with a medium-long (15 to 20 cm), light green petiole. The Leychoi type has shiny, dark green, convex leaves with a thick, white petiole. The Joychoi type has a small (18 to 20 cm), spreading or semi-spreading leaf rosette with slightly upright, very compact, fragile leaves [40].

Pak choi is a particularly common plant in central and southern China. In many industrially developed regions of the world, such as the USA, European Union countries, Singapore, Japan, China, and South Korea, due to the high demand for *Brassicaceae* plants and their rapid growth, *B. rapa* var. *chinensis* is cultivated hydroponically outside of traditional field or greenhouse cultivation methods [40,41]. Water shortages and low UV radiation intensity can cause a decrease in biomass and photosynthetic capacity [42]. Therefore, vertical farming (VF) is a good solution, resembling a warehouse with multiple levels of shelves and creating a closed cultivation system. Such cultivation is carried out under artificial light, is air-conditioned, is circulated, and is supplied with carbon dioxide and nutrient solution. It is unaffected by unfavorable environmental conditions. It guarantees uninterrupted vegetable production year-round, using minimal water consumption. It has been demonstrated that the vertical hydroponic cultivation system resulted in more than three times greater production efficiency of *B. rapa* var. *chinensis* per square meter [42,43].

In traditional cultivation, organic and inorganic fertilizers are used. Due to concerns about the excessive use of inorganic fertilizers and their environmental impact, organic fertilizers are more frequently used. Recommended fertilizers include nitrogen, which is essential for the biosynthesis of nucleic acids, proteins, and amino acids; calcium, which ensures the integrity and permeability of the cell membrane; potassium, which plays a key role in plant metabolism; phosphorus, which affects root development and energy storage; and magnesium, which influences chlorophyll production [44]. Pak choi is a popular plant grown in tropical regions because it is harvested 40–60 days after sowing. Cultivation costs are low. Seeds are easy to obtain, and the plant adapts readily to tropical conditions [45].

Pak choi has a short growing season, and in Polish climatic conditions, it is harvested from September to October. The production technique is similar to that of Chinese cabbage. Good seedling quality is important during cultivation, as it influences the further growth and development of the plant in the field. Research indicates a correlation between later seedling preparation and higher yields. Furthermore, it has been found that delayed production affects the increase in fresh weight of the seedlings, but causes a decrease in dry weight, carotenoids, and simple sugars in the harvested vegetables [46].

**Figure 2:**
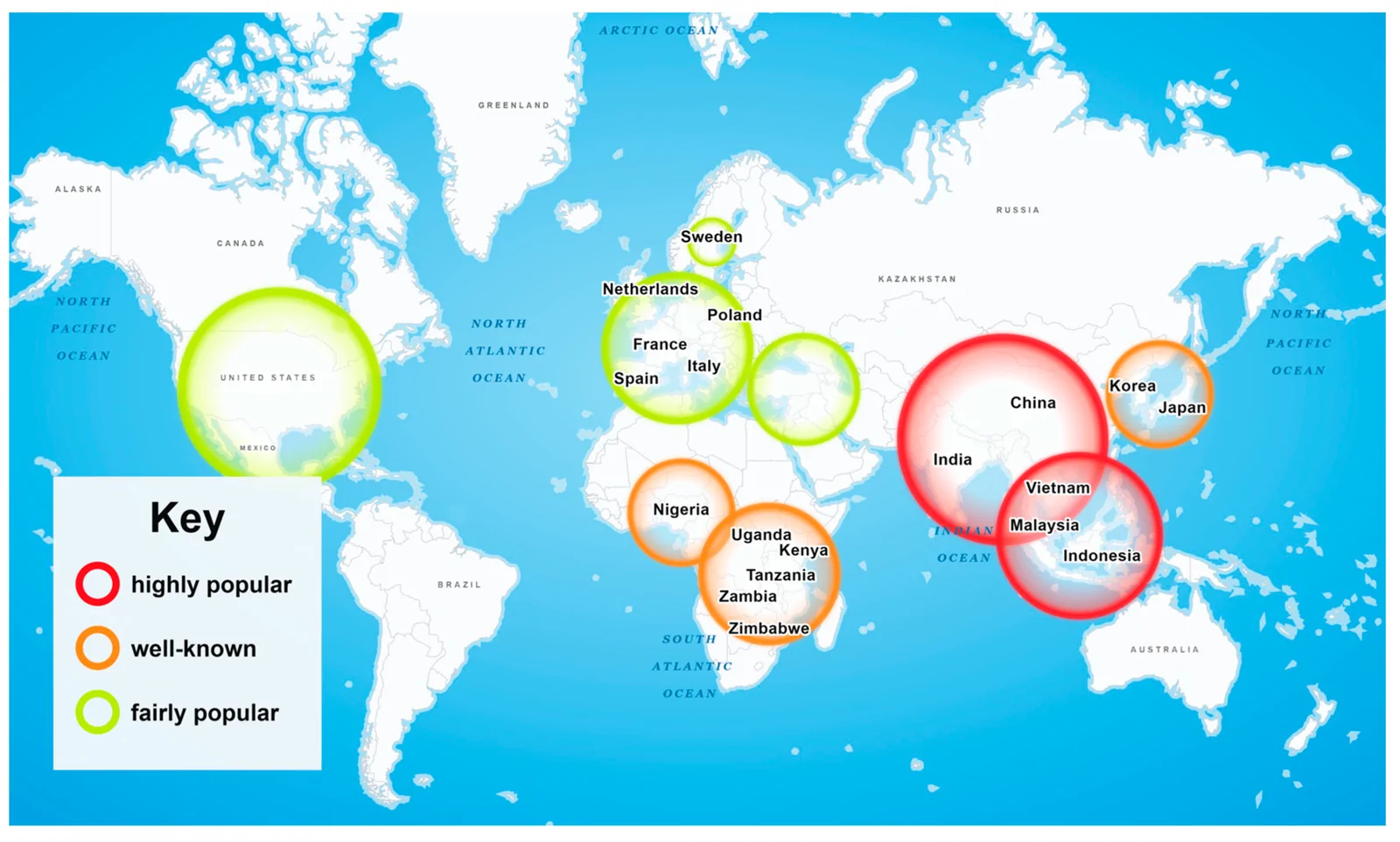
Global distribution and consumption of *B.rapa* subsp. *chinensis*.

In Asia, including China, Korea, Vietnam, Malaysia, India, Japan, and Indonesia, *Brassica rapa* L. subsp. *chinensis* constitutes an important component of the daily diet [5,6,8]. Among these populations, pak choi is widely preferred compared with other leafy vegetables and remains a staple food in many regions (Figure 2).

In Africa, particularly in countries such as Kenya, Tanzania, Nigeria, Uganda, Zambia, and Zimbabwe, pak choi contributes to local diets and food security. A portion of the production is also directed toward export markets [12].

In Europe and North America, including countries such as the Netherlands and Sweden, *B. rapa* subsp. *chinensis* has gained increasing popularity as a culinary ingredient, being incorporated into a variety of modern dishes [10,13]. The vegetable is typically consumed after thermal processing, such as stir-frying, blanching, or boiling, whereas raw consumption remains relatively uncommon, particularly in culinary traditions derived from East Asia [6].

The use of the *chinensis* subspecies originated in China, where it was cultivated earlier than *B. rapa* subsp. *pekinensis*, and subsequently disseminated worldwide, contributing to its current global distribution.

The *Brassicaceae* family is of considerable economic importance worldwide, providing raw materials for oil production, condiments, and fresh vegetables with significant nutritional value for both humans and animals [1,3,5]. Increasing demand for plant-based and organic foods has contributed to the intensification of agricultural production [20].

It has been estimated that approximately one in ten vegetable farms globally cultivates cruciferous species [14]. Earlier data indicated that global production of *Brassicaceae* vegetables exceeded 600 kilotons per year, with a clear upward trend [6]. More recent reports confirm a substantial increase in production, reaching over 55,146 kilotons in 2021 [47].

The growing popularity of pak choi is further supported by its favorable agronomic characteristics, including a short cultivation cycle—allowing harvest as early as approximately 30 days after sowing—adaptability to relatively low temperatures, and low soil requirements. In addition, its mild and widely acceptable flavor contributes to high consumer acceptance [10,14].

These factors have significantly contributed to the economic and commercial importance of *B. rapa* subsp. *chinensis* in Asia, where it has been cultivated continuously for over 1000 years, particularly in coastal regions [8].

Among cruciferous vegetables, pak choi is one of the most widely cultivated species, accounting for approximately 30–40% of vegetable production areas in China. Cultivation is predominantly concentrated in East Asia, with green-petiole varieties more common in northern regions and white-petiole types prevailing in southeastern areas [7]. In addition to open-field cultivation, *B. rapa* subsp. *chinensis* is extensively grown in controlled environments, including greenhouse systems, allowing for stable and efficient production [15].

Seasonal variation in cultivation practices further contributes to the diversity of pak choi production. Distinct varieties are adapted to different sowing periods, including summer, autumn, winter, and spring cycles, enabling year-round availability, particularly in regions with favorable climatic conditions [7].

The long-standing cultivation of pak choi in Asia reflects its sustained dietary and agronomic importance, whereas in Europe its cultivation and consumption have progressively increased over the past several centuries [9].

## 5. Status as a Superfood and Dietary Stats

The term “superfood” is widely employed in nutritional science and public discourse to describe plant-derived foods with enhanced health-promoting properties; however, it lacks a formal definition and is not recognized by regulatory bodies such as the World Health Organization (WHO), the Food and Agriculture Organization (FAO), or the European Food Safety Authority (EFSA) [48]. Despite this limitation, the concept fully persists in scientific literature due to the well-documented high content of micronutrients and bioactive compounds in such foods [17]. In contrast, the concept of “functional food” is more accepted, although its interpretation remains flexible and potentially subject to misapplication in both scientific and commercial contexts [17,27].

**Figure 3:**
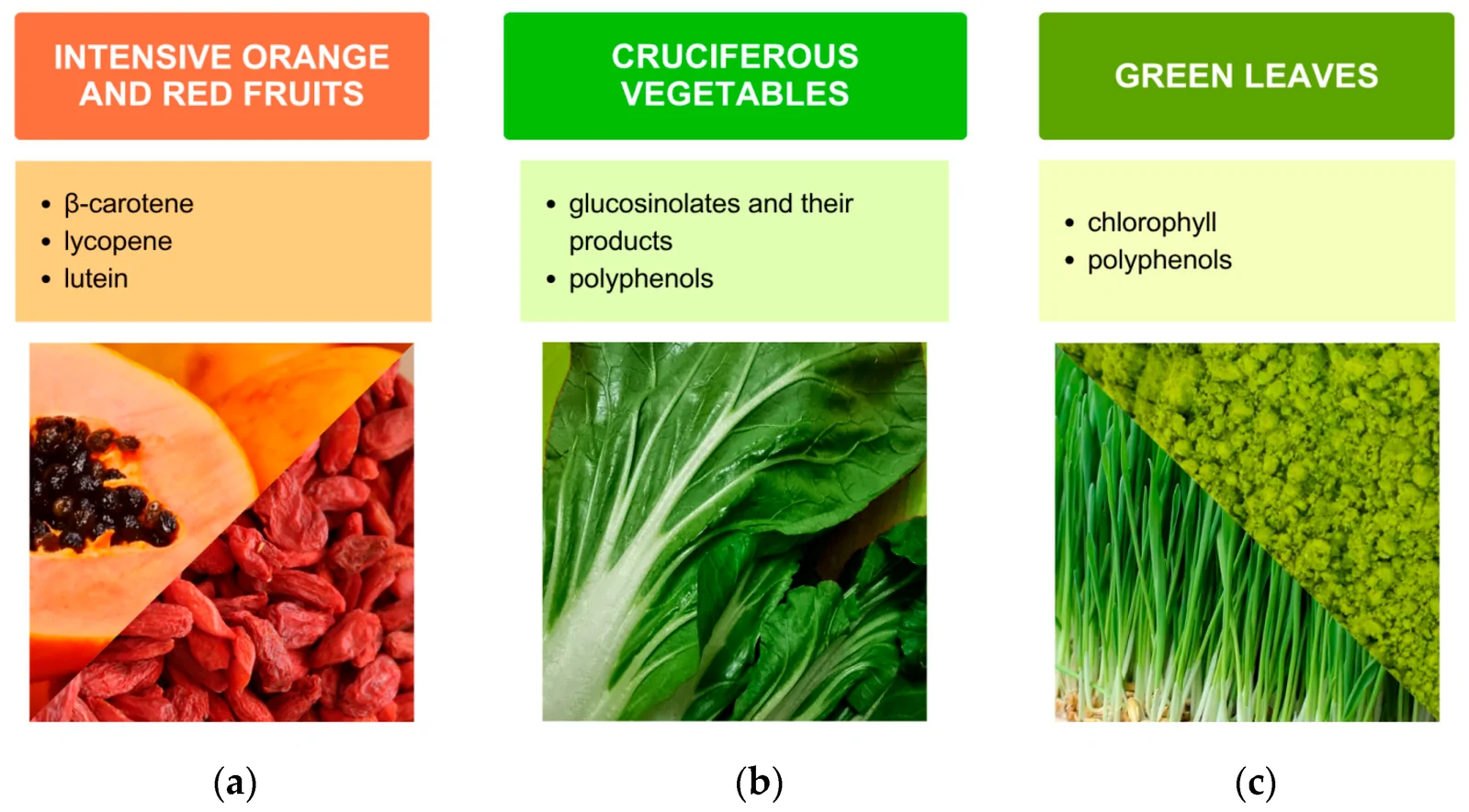
Classification and representation of superfoods: (**a**): Intensive orange and red fruits—representatives: papaya and goji berries; (**b**): Cruciferous vegetables—representative: pak choi; (**c**): Green leaves—representatives: barley grass and matcha green tea.

Vegetables regarded as “superfoods” typically belong to three dominant groups: intensely pigmented fruits and vegetables rich in carotenoids (e.g., β-carotene, lycopene), *Brassicaceae* species distinguished by the presence of glucosinolates and their biologically active hydrolysis products—primarily isothiocyanates—along with polyphenols, micronutrients, and leafy green vegetables characterized by high chlorophyll, fiber, and mineral content [49]. Within this framework, *B. rapa* subsp. *chinensis* can be classified as a functional food candidate due to its combined nutritional and phytochemical characteristics (Figure 3).

Despite the fact that *B. rapa* subsp. *chinensis* is not formally noted as a “superfood”, its nutrient and phytochemical profile very closely aligns with the criteria associated with this category: the species is characterized by high levels of vitamins, minerals, and dietary fiber, combined with a low caloric value, and a rich pool of secondary metabolites, including flavonoids and thioglycosides [1,30]. In particular, glucosinolates and their hydrolysis products have been extensively studied as key mediators of the health-promoting potential of *Brassicaceae* vegetables [50].

Nevertheless, it should be emphasized that the classification of plant foods as “superfoods” often oversimplifies their biological complexity. Advanced metabolomic analyses of *B. rapa* subsp. *chinensis* have revealed hundreds of metabolites, including phenolic acids, flavonoids, amino acids, and glucosinolate derivatives, with significant differences among cultivars and growth stages [16,51]. This variability directly influences the nutritional value and biological activity of the plant, highlighting the need for a more nuanced and evidence-based approach to its classification and utilization.

*B. rapa* subsp. *chinensis* is characterized by high nutritional density, making it a representative example of plant-based “superfoods”. Fresh plant material contains elevated levels of micronutrients and oxidation-sensitive compounds, which are best preserved when consumed shortly after harvest [20]. Particularly, young shoots and microgreens of *B. rapa* subsp. *chinensis* are considered valuable dietary components due to their enhanced phytochemical activity and contribution to health-promoting dietary patterns [52,53].

The chemical composition of *B. rapa* subsp. *chinensis* reflects both primary nutritional value and functional properties. The plant is composed of approximately 76–92.5% water, contributing to hydration and metabolic homeostasis [7]. It is additionally a significant source of dietary fiber, including soluble pectins and insoluble fractions such as cellulose, hemicellulose, and lignin, which regulate gastrointestinal motility and glucose metabolism [11,24]. Quantitative analyses indicate that per 100 g of fresh weight, the plant provides approximately 2.7 g of fiber, 3.1 g of carbohydrates, 1.7 g of protein, and trace amounts of fat, alongside essential micronutrients such as vitamin C (53 mg), calcium (102 mg), and provitamin A (2.3 mg β-carotene) [6,11] (Table 4).

Meta-analytical data indicate that regular consumption of fruits and vegetables reduces the risk of metabolic and cardiovascular diseases, with dose-dependent effects observed at intake levels of 200–800 g per day [49]. From a health perspective, regular consumption of *B. rapa* subsp. *chinensis* contributes to multiple physiological benefits, including improved hydration, regulation of digestive processes, stabilization of blood glucose levels, and reduction in oxidative stress through the activity of vitamins and plant pigments [20,49]. Constituents in pak choi are associated with the modulation of oxidative stress and inflammatory pathways, thereby contributing to the prevention of non-communicable diseases, including cardiovascular and metabolic disorders [1,30].

Diets rich in vegetables with high phytochemical content have been associated with reduced incidence of chronic diseases, like gastrointestinal cancers [63]. These include polyphenols, carotenoids, and sulfur-containing compounds, which are known to exert antioxidant, anti-inflammatory, and chemoprotective effects [17,29].

Public awareness of the health benefits of functional and nutrient-dense plant foods remains heterogeneous across populations. European surveys indicate that a considerable proportion of consumers either lack familiarity with or express skepticism toward the concept of “superfoods” [17]. Consumer preferences are additionally shaped by socio-economic determinants, including income, education level, and product pricing, which influence both accessibility and perception of functional foods [17,21]. While interest in exotic, imported products remains high, there is a parallel trend favoring locally available plant foods with comparable nutritional value. This phenomenon supports the growing importance of widely accessible *Brassicaceae* vegetables, including *B. rapa* subsp. *chinensis*, as components of sustainable and health-oriented dietary patterns [64].

During the uncomplicated and low-maintenance cultivation of microgreens, the germination process releases and significantly increases the concentration of wholesome constituents from the seed material, along with stronger antioxidant activity compared to mature plants. This indicates their potential as highly valuable functional food ingredients [65]. The global market for microgreens and edible sprouts has expanded dynamically, reflecting increasing demand for concentrated sources of bioactive compounds. *Brassicaceae* species represent a substantial proportion of this sector, particularly within the European Union [66].

These findings position *B. rapa* subsp. *chinensis* as a plant of high nutritional and functional relevance. However, the lack of standardized classification criteria, coupled with variability in phytochemical composition and bioavailability, underscores the need for further integrative research to fully define its role within modern evidence-based nutrition.

*B. rapa* subsp. *chinensis* exhibits considerable variability in primary metabolite profiles, with metabolomic analyses identifying dozens of compounds across different cultivars [16]. This variability extends to nitrogen metabolism, where the content of nitrate ions (NO_3_^−^) and total nitrogen differs significantly depending on cultivar, developmental stage, and environmental conditions [24,25]. Importantly, nitrogen-containing primary metabolites play a main role in plant metabolism, serving as precursors for biologically active secondary compounds.

In particular, amino acids present in *B. rapa* subsp. *chinensis* are not only relevant from a nutritional standpoint but also constitute key intermediates in the biosynthesis of secondary metabolites characteristic of the *Brassicaceae* family. Compounds such as methionine, phenylalanine, and tryptophan serve as precursors for glucosinolate biosynthesis, linking primary metabolic pathways with bioactive compound formation [67,68,69]. This metabolic interdependence highlights the importance of considering both primary and secondary metabolites in evaluating the functional properties of plant-derived foods.

Evolving market trends further support the growing importance of *B. rapa* subsp. *chinensis* in modern nutrition. The expansion of the microgreens and sprouts sector, particularly within Europe, reflects increasing demand for plant foods with concentrated bioactive profiles. Taken together, these findings confirm that pak choi fulfills key criteria associated with nutrient-dense and health-promoting foods. However, its classification should be approached within a scientifically grounded framework that accounts for variability in composition, bioavailability and cultivation conditions, rather than relying solely on simplified terminology such as “superfood”.

## 6. Chemical Composition

The chemical composition of *B. rapa* subsp. *chinensis* has been extensively characterized using advanced analytical techniques, including gas chromatography–mass spectrometry (GC–MS), inductively coupled plasma mass spectrometry (ICP–MS), ultra-high-performance liquid chromatography coupled with tandem mass spectrometry (UHPLC–MS/MS), and electrospray ionization (ESI) [3,9,16,70]. These approaches have enabled comprehensive profiling of both primary and secondary metabolites.

**Figure 4:**
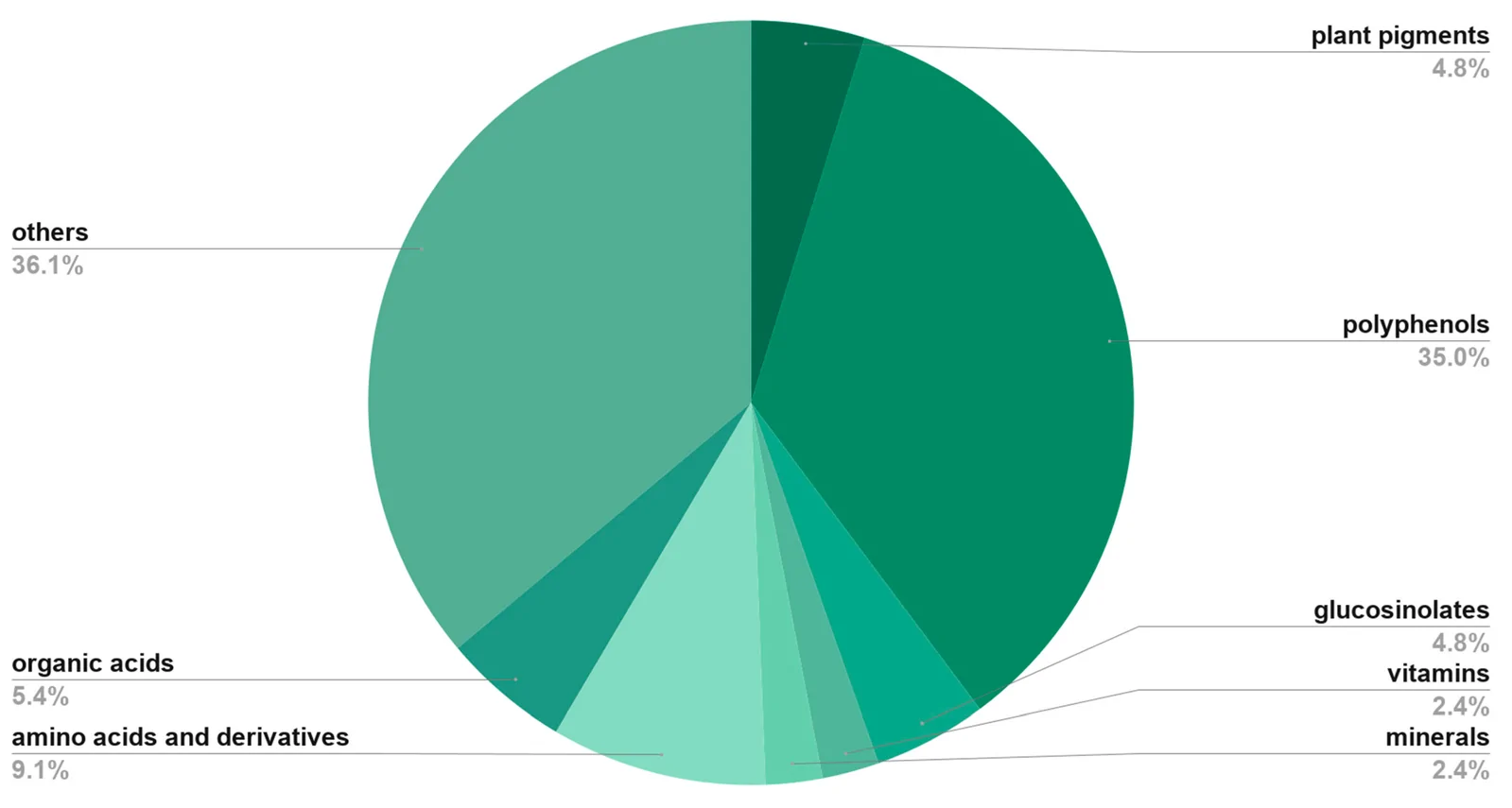
Relative contribution of major classes of primary and secondary metabolites identified in leaves of *B. rapa* subsp. *chinensis* based on comprehensive metabolomic profiling.

The phytochemical profile of *B. rapa* subsp. *chinensis* shows considerable intra- and interspecific variability and is influenced by genotype, developmental stage, morphological traits, and environmental conditions [2,71,72]. Quantitative analyses indicate an ash content ranging from 7.5% to 24% of dry weight [7]. Metabolomic studies have identified numerous compound classes, including alkaloids (7.31%), amino acids and their derivatives (9.10%), organic acids (5.37%), lipids (15.52%), phenolic acids (19.85%), flavonoids (14.93%), glucosinolates (4.78%), lignans and coumarins (5.07%), carbohydrates (5.22%), vitamins (2.39%), and terpenoids (1.34%). In total, up to 670 metabolites were detected across the cultivars ‘Qianyecai’ (‘QYC’), ‘Maertou’ (‘MET’), ‘Naibaicai’ (‘NBC’), ‘Paopaoqing’ (‘PPQ’), and ‘Xiangqingcai’ (‘XQC’), demonstrating the considerable chemical diversity of the species [16] (Figure 4).

Among the major bioactive constituents, glucosinolates represent a characteristic group of sulfur-containing secondary metabolites typical of *Brassicaceae* [48,71,72]. Pak choi is also a rich source of polyphenolic compounds, particularly flavonoids and phenolic acids, as well as anthocyanins and carotenoids, including lutein [16,28,31]. Organic acids, particularly citric and malic acids, together with essential minerals and vitamin C, further contribute to its nutritional and functional value [7,9].

Importantly, this composition is not constant. Environmental and agronomic factors, including temperature, light intensity, soil properties, irrigation, and fertilization, can modify the phytochemical profile, resulting in variation among geographical origins and cultivation systems [70]. Post-harvest handling, storage, transport, and processing may further affect the retention and stability of bioactive constituents [24]. Consequently, the substantial chemical diversity of *B. rapa* subsp. *chinensis* reflects the combined influence of genotype, developmental stage, cultivation conditions, and post-harvest factors and provides the basis for the detailed discussion of its major phytochemical groups in the following sections.

### 6.1. Polyphenolic Compounds

In *B. rapa* subsp. *chinensis*, polyphenolic compounds are present in diverse amounts, contributing to both nutritional value and biological activity. Within green cultivars, total phenolic content values vary considerably. Light-colored cultivars typically exhibit lower levels—164.5 µg·g^−1^ DW, whereas dark cultivars and those with purple hues reach higher levels—548.6 µg·g^−1^ DW, although these relationships extend beyond primary genetics to include developmental stages and environmental conditions [60]. The total phenolic content has been estimated at approximately 2170 µg·g^−1^ gallic acid equivalents (GAE) fresh weight (FW) in selected cultivars, such as ‘Pak choi White’, which are cultivated in Europe (Romania) [73]. In microgreens, the concentration of phenolics reaches approximately 2000–5000 µg·g^−1^ DW, which reflects the enhanced metabolic activity at early developmental stages [66]. In contrast, anthocyanin accumulation in *B. rapa* subsp. *chinensis* is generally low, indicating that this species is not a major dietary source of these pigments compared with other *Brassica* taxa [9].

The major classes of phenolic compounds identified in *B. rapa* subsp. *chinensis* derive predominantly from phenylalanine via the phenylpropanoid pathway, which represents a central metabolic route linking primary and secondary plant metabolism [16]. These compounds are commonly divided into hydroxycinnamic acid derivatives, including caffeic acid, ferulic acid, sinapic acid, chlorogenic acid, and rosmarinic acid, and hydroxybenzoic acid derivatives, such as gallic acid and protocatechuic acid [74] (Table 5).

Detailed metabolomic analyses have confirmed the presence of a wide range of phenolic compounds in different cultivars of *B. rapa* subsp. *chinensis*, with considerable variability depending on genetic background. Among the most frequently detected compounds are chlorogenic acid, caffeic acid, ferulic acid, sinapic acid, and their derivatives, as well as flavonoid glycosides (Table 5). Differences between cultivars have been observed, for example, with higher accumulation of sinapic acid and related derivatives in ‘Qianyecai’ (QYC) compared with ‘Xiangqingcai’ (XQC) [16].

Notably, certain compounds have been reported for the first time in *B. rapa* subsp. *chinensis*, including rosmarinic acid-3′-O-glucoside (syringin), as well as isoferulic and isochlorogenic acids, reflecting the increasing resolution of modern metabolomic techniques. Additionally, cultivar-specific occurrence of compounds such as protocatechuic acid in ‘Maertou’ (MET) highlights the importance of genetic factors in shaping phenolic profiles [16].

The presence and relative abundance of these compounds contribute significantly to the antioxidant potential of *B. rapa* subsp. *chinensis*, as well as its role in modulating oxidative stress and inflammatory pathways. Importantly, the variability in polyphenol composition among cultivars and developmental stages indicates that quantitative and qualitative differences should be considered when evaluating the nutritional and functional properties of this species.

### 6.2. Flavonoids

Flavonoids represent one of the most important subclasses of polyphenolic compounds identified in *B. rapa* subsp. *chinensis*. This group includes structurally diverse compounds such as flavonols, flavanones, flavones, flavanols, flavanonols, anthocyanins, and chalcones, which differ in both chemical structure and biological activity [16,69]. Among these, flavonols constitute the dominant fraction, accounting for approximately 53% of the total flavonoid pool in this species [16].

Quantitative analyses indicate that the total flavonoid content in *B. rapa* subsp. *chinensis* may reach approximately 124.2 mg quercetin equivalents (QE) per 100 g FW, as demonstrated for the ‘Pak choi White’ cultivar [73]. In comparison with other *Brassicaceae* vegetables, flavonoids represent a major group of secondary metabolites, contributing up to 35% of the total phytochemical composition [75].

Detailed metabolomic investigations have identified a broad spectrum of flavonoid compounds in *B. rapa* subsp. *chinensis*, including kaempferol, quercetin, isorhamnetin and numerous glycosylated derivatives (Table 6). Among these, kaempferol is typically the most abundant compound in green-leaved cultivars, followed by isorhamnetin and quercetin, along with their respective derivatives [76]. Glycosylated forms, such as kaempferol-3,7-di-O-glucoside and isorhamnetin-3,7-di-O-glucoside, are also frequently detected and contribute to the overall diversity of flavonoid profiles.

The composition of flavonoids is strongly influenced by cultivar type and phenotypic traits, particularly leaf coloration. Red and purple varieties of *B. rapa* subsp. *chinensis* have been shown to accumulate significant levels of anthocyanins, especially delphinidin 3-O-glucoside, whereas green cultivars are characterized by a predominance of flavonols [77]. In total, up to 92 different flavonoid compounds have been identified across various cultivars, highlighting the considerable chemical diversity within this species.

Comparative analyses further indicate that *B. rapa* subsp. *chinensis* shares a flavonoid profile similar to other *Brassicaceae* species, such as *Brassica juncea*, particularly with respect to the dominance of kaempferol- and isorhamnetin-based glycosides [9]. This similarity reflects conserved metabolic pathways within the family, while cultivar-specific differences are driven by genetic and environmental factors.

Importantly, the accumulation of flavonoids is closely associated with antioxidant capacity, as these compounds act as effective scavengers of reactive oxygen species and modulators of oxidative stress pathways [16]. Therefore, their presence contributes significantly to the biological activity and functional value of *B. rapa* subsp. *chinensis*, reinforcing its relevance in health-oriented nutrition.

### 6.3. Glucosinolates

Glucosinolates are a characteristic group of sulfur- and nitrogen-containing secondary metabolites specific to the *Brassicaceae* family. Their biosynthesis originates from amino acids, which serve as structural precursors forming the variable side chain of the molecule, while the core structure consists of a β-thioglucose moiety linked to a sulfonated oxime group [68,79]. The presence of sulfur and nitrogen is essential for the formation of glucosinolates, and their accumulation is often intensified under environmental stress conditions, reflecting their role in plant defense and adaptation [66,69,80].

To date, approximately 140 glucosinolate compounds (thioglycosides) have been identified in plants, exhibiting a wide range of chemical structures and biological activities [48,68,69,81]. Based on the structure of the amino acid-derived side chain, glucosinolates are classified into three main groups: aliphatic, aromatic, and indolic compounds [48,82]. Their content is highly variable and depends on genetic background, developmental stage, and cultivation conditions, and is typically expressed in µmol per FW or DW (dry weight) [83].

**Figure 5:**
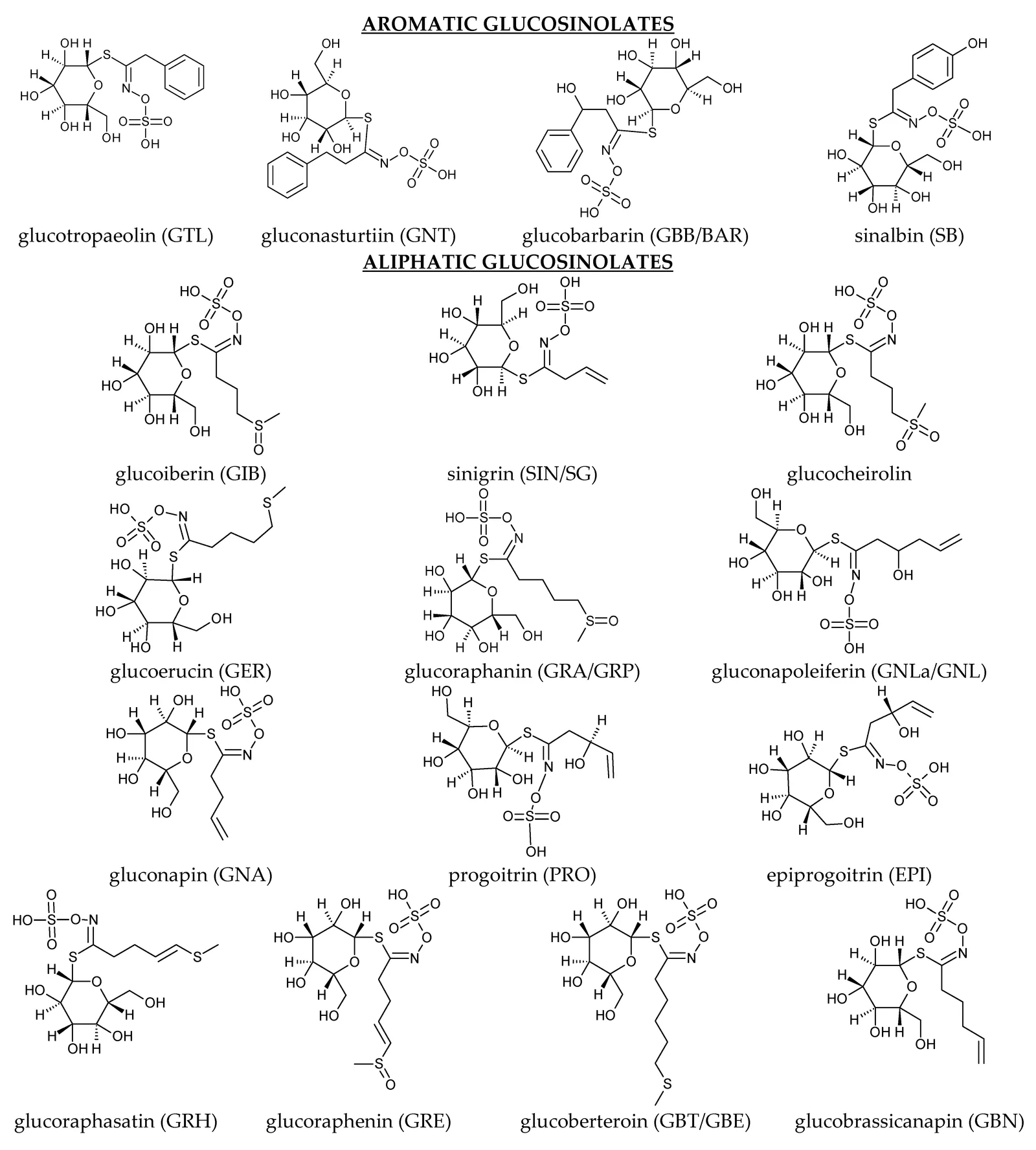

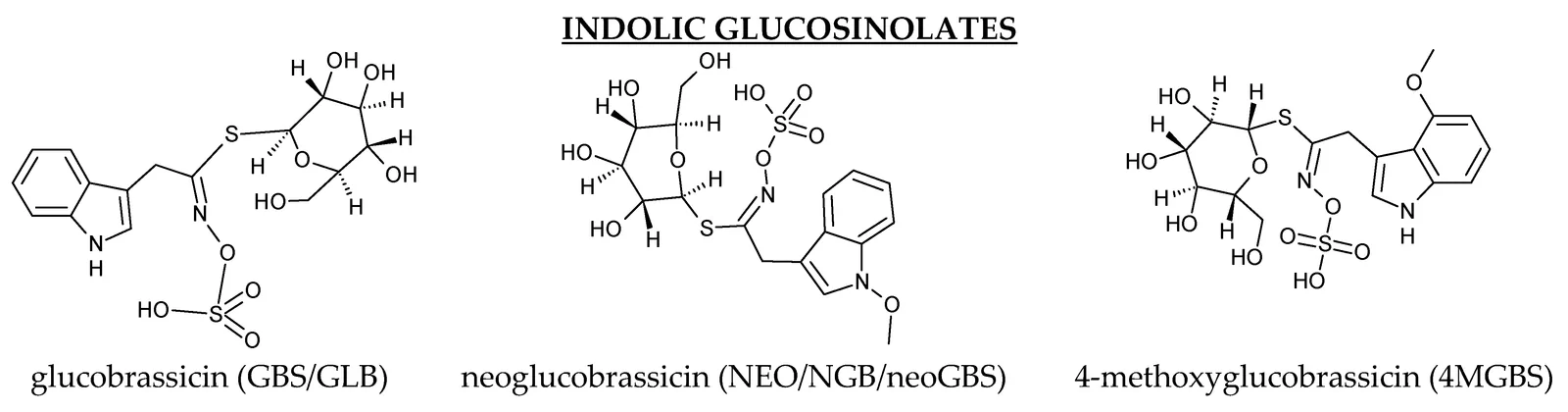
Chemical formulas of glucosinolates identified in *B. rapa* subsp. *chinensis*, grouped by category.

In *B. rapa* subsp. *chinensis*, up to 17 individual glucosinolates have been identified, with aliphatic compounds representing the dominant fraction [34,68,69,83] (Figure 5). The total glucosinolate content in the leaves depends on the developmental stage, and high levels, such as 9.1 μmol∙g^−1^ FW have been identified especially in young leaves [84]. Significantly higher levels may accumulate in roots, reaching up to 283 μmol∙g^−1^ FW, which is approximately 31 -fold higher than in aboveground tissues [85].

Variability in glucosinolate content has also been demonstrated among cultivars and under different environmental conditions. For example, plants with green petioles cultivated under adequate water availability exhibited average glucosinolate levels of 23 μmol∙g^−1^ FW, compared with 17 μmol∙g^−1^ FW in white-petiole forms. Among analyzed cultivars, ‘Shanghai Late May’ showed the highest content (38.2 μmol∙g^−1^ FW), whereas ‘KSL Nylon Pakchoi’ and ‘Early Huang-Jing Pakchoi’ exhibited the lowest values (6.9 μmol∙g^−1^ FW) [7]. Similarly, metabolomic studies identified ‘Qianyecai’ and ‘Xiangqingcai’ as cultivars with relatively high glucosinolate content, while ‘Naibaicai’ exhibited lower levels [16].

Detailed qualitative and quantitative data for individual glucosinolates are presented in Table 7. Among aliphatic glucosinolates, the most abundant compounds include gluconapin (up to 19,009.896 μmol∙kg^−1^ DW; average 6713.083 μmol∙kg^−1^ DW) and glucobrassicanapin (up to 8744.337 μmol∙kg^−1^ DW; average 3139.729 μmol∙kg^−1^ DW), followed by progoitrin (up to 4116.955 μmol∙kg^−1^ DW; average 1132.364 μmol∙kg^−1^ DW) and epiprogoitrin (up to 3333.335 μmol∙kg^−1^ DW; average 843.059 μmol∙kg^−1^ DW) [34]. Other compounds within this group include glucoraphanin (average 172.591 μmol∙kg^−1^ DW), glucoerucin (average 49.366 μmol∙kg^−1^ DW), and glucoberteroin (average 148.188 μmol∙kg^−1^ DW). Minor constituents include glucoraphasatin (average 0.231 μmol∙kg^−1^ DW), glucoiberin (average 0.375 μmol∙kg^−1^ DW), glucoraphenin (average 0.981 μmol∙kg^−1^ DW), and glucocheirolin (average 5.256 μmol∙kg^−1^ DW). In the aromatic group, gluconasturtiin is one of the most abundant compounds (74.282–2148.237 μmol∙kg^−1^ DW; average 678.72 μmol∙kg^−1^ DW), whereas sinalbin occurs at very low levels (0–3.704 μmol∙kg^−1^ DW; average 0.086 μmol∙kg^−1^ DW) [34]. Within indolic glucosinolates, glucobrassicin is present at 77.984–1294.483 μmol∙kg^−1^ DW (average 351.011 μmol∙kg^−1^ DW), while neoglucobrassicin and 4-methoxyglucobrassicin occur at lower concentrations [13,34].

Sprouts and microgreens of *B. rapa* subsp. *chinensis* exhibit particularly high glucosinolate concentrations, with progoitrin reaching approximately 17.84 mg∙g^−1^ DW, gluconapin 4.5 mg∙g^−1^ DW, and glucobrassicanapin 1.96 mg∙g^−1^ DW [86]. These values indicate a higher accumulation of certain bioactive compounds in early developmental stages compared with mature tissues.

Among individual compounds, neoglucobrassicin is considered characteristic for this species, although profiling based solely on glucosinolate composition may not reliably indicate geographical origin due to high variability in biosynthesis [70,85].

Overall, aliphatic glucosinolates represent the predominant class in *B. rapa* subsp. *chinensis*, followed by aromatic and indolic compounds, although in certain conditions indolic glucosinolates may reach comparable levels [34,66]. The high abundance and diversity of glucosinolates, particularly gluconapin, confirm the importance of this species as a rich source of biologically active sulfur-containing compounds.

**Figure 6:**
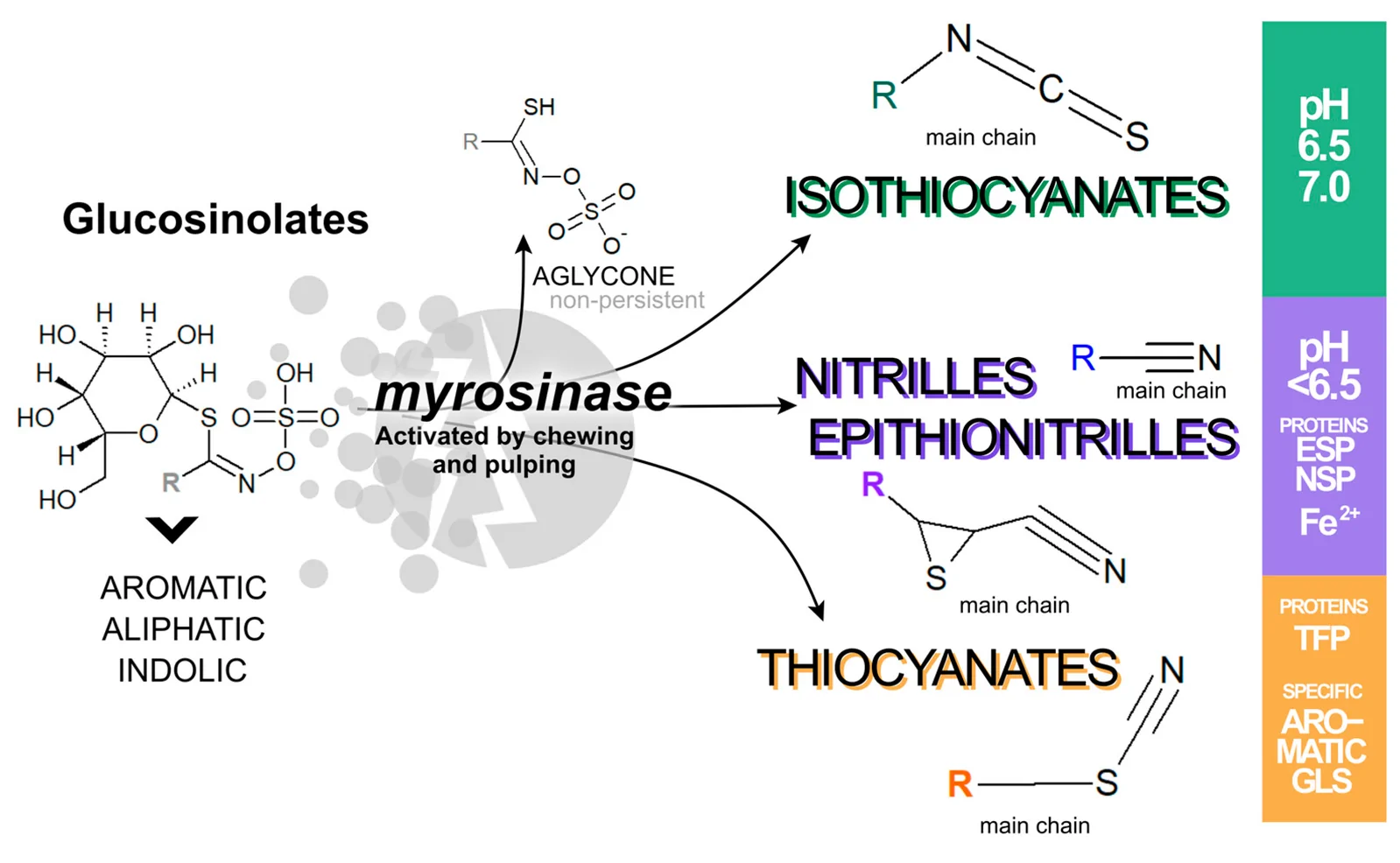
The process of glucosinolate transformation involving myrosinase and varying environmental reaction conditions.

Structural disruption of *B. rapa* subsp. *chinensis* tissues, resulting from mechanical damage, processing or senescence, plays a crucial role in the conversion of glucosinolates into biologically active compounds, primarily isothiocyanates [80] (Figure 6). This transformation is catalyzed by myrosinases (β-thioglucosidases), which are compartmentalized separately from glucosinolates in intact plant tissues and become enzymatically active only upon cell disruption. Mechanical factors such as cutting, crushing, or chewing enable the interaction between enzyme and substrate, initiating hydrolysis and the formation of new bioactive metabolites. Under undisturbed conditions, when plant tissues remain intact, myrosinases and glucosinolates are spatially separated, and hydrolysis does not occur. This structural compartmentalization represents a defensive strategy in plants, allowing rapid activation of chemical defense mechanisms after tissue damage.

In addition to isothiocyanates, glucosinolate hydrolysis may yield a variety of other products, including thiocyanates, nitriles, sulfates, amines, indole alcohols and glucose [69]. The direction of these transformations is influenced by multiple factors, such as pH, the presence of metal ions (e.g., Fe^2+^), environmental conditions, and the chemical structure of the parent glucosinolate [68]. Representative glucosinolate-derived products identified in *B. rapa* subsp. *chinensis* are summarized in Table 8.

Isothiocyanates are typically formed through enzymatic hydrolysis of glucosinolates, while alternative products such as epithionitriles may arise through subsequent cyclization reactions involving intermediate isothiocyanates. In particular, glucosinolates such as sinigrin, gluconapin, gluconapoleiferin, and glucobrassicanapin may undergo structural rearrangements under specific conditions, leading to the formation of cyclic sulfur-containing derivatives, including epithionitriles [87].

Quantitative studies indicate that the total concentration of glucosinolate-derived hydrolysis products in *B. rapa* subsp. *chinensis* may reach approximately 139.2 µmol·g^−1^ DW following enzymatic conversion by myrosinases [28]. These compounds are of particular interest in biomedical research due to their well-documented antitumor activity observed in experimental models, including studies on cancer cell lines [3].

### 6.4. Plant Pigments

The green coloration of *B. rapa* subsp. *chinensis* reflects a high content of chlorophylls, which are the primary photosynthetic pigments responsible for light absorption and energy conversion in plants [88]. Due to its soft tissue structure and large leaf surface, *B. rapa* subsp. *chinensis* is particularly susceptible to post-harvest water loss and subsequent pigment degradation. Nevertheless, the species is characterized by relatively high chlorophyll content [89]. Cultivation practices, especially under controlled conditions such as hydroponic systems, may significantly influence pigment accumulation. For instance, the application of osmotic regulators under reduced nitrate levels has been shown to enhance chlorophyll content, improving both visual quality and sensory attributes [90].

In addition to chlorophylls, carotenoids represent an important group of plant pigments in *B. rapa* subsp. *chinensis*. These tetraterpenoid compounds (C_40_) are synthesized via the isoprenoid pathway and perform multiple biological functions, including light harvesting, protection of the photosynthetic apparatus and serving as precursors for signaling molecules [91,92]. Carotenoids are lipophilic compounds and include two major subclasses: carotenes (e.g., α-carotene, β-carotene, lycopene) and xanthophylls (e.g., lutein, zeaxanthin, neoxanthin, violaxanthin), which differ by the presence of oxygen functional groups [93,94].

In *B. rapa* subsp. *chinensis*, lutein represents the dominant carotenoid, accounting for approximately 40–43% of the total carotenoid pool, followed by violaxanthin (approximately 28%) and neoxanthin (approximately 13%). Carotenoid accumulation is influenced by environmental factors, particularly light intensity, with higher concentrations observed in well-illuminated outer leaves. Under drought conditions, carotenoid levels may increase, with reported values up to approximately 25% higher compared with well-watered plants [7].

Anthocyanins, belonging to the flavonoid group, represent an additional class of pigments present predominantly in purple cultivars of *B. rapa* subsp. *chinensis*. These compounds accumulate mainly in epidermal tissues and include derivatives such as delphinidin 3-O-glucoside and related anthocyanins [77,95]. In green cultivars, anthocyanins are absent or present only in trace amounts, whereas in purple varieties they may constitute up to approximately 50% of total flavonoids [77]. Maximum reported anthocyanin concentrations reach 735.39 mg·kg^−1^ under optimized lighting conditions, while typical values in microgreens range from 0.1 to 0.3 mg·g^−1^ DW [66,96].

A positive correlation has been observed between carotenoid content and chlorophyll *a* and *b*, as well as between total phenolic content and chlorophyll *b* levels, suggesting coordinated regulation of pigment and secondary metabolite synthesis [73]. Furthermore, the distribution of pigments within the plant is uneven, with lower concentrations typically detected in inner leaves due to reduced light exposure [97].

Quantitative data summarised in Table 9 indicate clear differences between developmental stages. Microgreens generally exhibit lower pigment concentrations on a fresh weight basis, although exceptionally high values have been reported depending on cultivation conditions and analytical methodology [19]. In contrast, fully developed leaves display higher levels of chlorophyll *a* and *b* as well as total carotenoids, reflecting a more developed photosynthetic system [98]. Notably, total chlorophyll content (a + b) has been reported at 9646 µg·g^−1^ FW in microgreens, while β-carotene and lutein concentrations vary depending on growth stage and environmental conditions [19,62].

### 6.5. Amino Acids

*B. rapa* subsp. *chinensis* constitutes a valuable source of amino acids, including essential amino acids required in the human diet [32]. This amino acid profile includes multiple proteinogenic amino acids, as summarized in Table 10. Analytical data indicate that valine exhibits the highest signal intensity (15.82 ratio/g), followed by isoleucine (10.66 ratio/g) and threonine (9.22 ratio/g), whereas methionine (2.25 ratio/g) and tryptophan (1.12 ratio/g) occur at lower levels. Intermediate values were recorded for alanine (6.42 ratio/g), phenylalanine (6.06 ratio/g), and leucine (7.84 ratio/g) [100].

Comparative metabolomic analyses across different cultivars (‘Maertou’, ‘Paopaoqing’, ‘Xiangqingcai’, ‘Qianyecai’, ‘Naibaicai’) revealed considerable variability in amino acid composition, including both protein-bound and free amino acids. Among the investigated cultivars, ‘Xiangqingcai’ showed the highest abundance of the majority of proteinogenic amino acids. The most intensive signals were observed for valine, tyrosine, and leucine, followed by isoleucine, phenylalanine, glutamic acid, and arginine, whereas serine, aspartic acid, and cysteine were present at relatively lower levels [16].

From an analytical perspective, precise quantification and digestibility of amino acids in plant-derived foods may be difficult to determine due to methodological differences and matrix effects. For this reason, both quantitative and qualitative profiling approaches are applied to assess their occurrence and biological relevance [101].

Importantly, amino acids play a crucial role not only as nutrients but also as key precursors of secondary metabolites characteristic of *Brassicaceae* plants. In particular, methionine, phenylalanine, and tryptophan are involved in the biosynthesis of glucosinolates, linking primary metabolic pathways with the formation of bioactive sulfur-containing compounds [67,68,69].

This interconnection between primary and secondary metabolism highlights the functional importance of amino acid composition in determining the overall bioactive potential of *B. rapa* subsp. *chinensis*. Variations in amino acid content are therefore directly related to fluctuations in secondary metabolite levels and, consequently, to the biological properties of the plant [31].

### 6.6. Vitamins and Minerals

*B. rapa* subsp. *chinensis* is considered a nutrient-dense vegetable, providing a broad spectrum of vitamins and minerals (Table 11 and Table 12), which contribute to its high nutritional value and relevance in the human diet [102,103]. This species contains vitamins A, K (phylloquinone), B-group vitamins and vitamin C, as well as essential minerals such as calcium, iron, potassium, magnesium, and zinc [11,16,24,54].

The content of fat-soluble vitamins varies depending on developmental stage and analytical approach. Vitamin A has been reported at 223 µg∙100 g^−1^ FW, while β-carotene concentrations in microgreens reached 854.7 mg∙kg^−1^ DW, corresponding to 71 mg RAE∙kg^−1^ DW [54,103]. Vitamin K levels in mature leaves were determined at 45.5 µg∙100 g^−1^ FW, whereas microgreens contained approximately 2.2 µg∙g^−1^ FW, indicating a substantially higher concentration on a mass basis [11,62].

Water-soluble vitamins are also present in moderate amounts, typical of green leafy *Brassicaceae* vegetables. Vitamin C content ranges from 45 mg∙100 g^−1^ FW in leaves to 56.9 mg∙100 g^−1^ FW in microgreens, while hydroponic cultivation systems have reported levels of 3.5–6.1 mg∙g^−1^ DW [10,62,103]. B-group vitamins, including B1, B2, B3, B5, B6, and B9, occur at moderate levels, although their distribution varies among cultivars and plant tissues [103]. Notably, vitamin B3 (niacin) has been detected in *B. rapa* subsp. *chinensis* at approximately 376.7 µg∙kg^−1^ FW and may occur at higher levels in purple cultivars compared with green forms (Table 11) [31,104].

**Table 11:** Content of vitamins in *B. rapa* subsp. *chinensis*.

**Fat-Soluble Vitamins**		
**Compound**	**Content**	**References**
Vitamin A	223 µg∙100 g^−1^ FW	[103]
	854.7 mg∙kg^−1^ DW β-carotene (= 71 mg RAE∙kg^−1^ DW) microgreen	[54]
Vitamin K	45.5 µg∙100 g^−1^ FW	[103]
	93.6 µg∙100 g^−1^ DW	[11]
	2.2 µg∙g^−1^ FW microgreen	[62]
**Water-soluble vitamins**		
Vitamin B_1_	0.04 mg∙100 g^−1^ FW	[103]
Vitamin B_2_	0.07 mg∙100 g^−1^ FW	[103]
Vitamin B_3_	0.5 mg∙100 g^−1^ FW	[103]
Vitamin B_5_	0.088 mg∙100 g^−1^ FW	[103]
Vitamin B_6_	0.194 mg∙100 g^−1^ FW	[103]
Vitamin B_9_	66 μg∙100 g^−1^ FW	[103]
Vitamin C	45 mg∙100 g^−1^ FW	[103]
	56.9 mg∙100 g^−1^ FW microgreen	[62]
	3.5–6.1 mg∙g^−1^ DW	[10]

RAE—Retinol Activity Equivalents.

**Table 12:** Content of minerals in *B. rapa* subsp. *chinensis*.

Minerals		
Compound	Content	References
Ferrum (Fe)	0.8 mg∙100 g^−1^ FW	[103]
	20.39–50.89 µg∙g^−1^ FW	[70]
	0.49 mg∙100 g^−1^ FW microgreen	[62]
Calcium (Ca)	105 mg∙100 g^−1^ FW	[103]
	5422–8268 µg∙g^−1^ FW	[70]
	58 mg∙100 g^−1^ FW microgreen	[62]
Potassium (K)	252 mg∙100 g^−1^ FW	[103]
	8683–12,113 µg∙g^−1^ FW	[70]
	284 mg∙100 g^−1^ FW microgreen	[62]
Magnesium (Mg)	19 mg∙100 g^−1^ FW	[103]
	518–629.6 µg∙g^−1^ FW	[70]
	31 mg∙100 g^−1^ FW microgreen	[62]
Zinc (Zn)	0.19 mg∙100 g^−1^ FW	[103]
	7.15–16.74 µg∙g^−1^ FW	[70]
	0.30 mg∙100 g^−1^ FW microgreen	[62]
Cuprum (Cu)	0.021 mg∙100 g^−1^ FW	[103]
	0.219–0.979 µg∙g^−1^ FW	[70]
	0.05 mg∙100 g^−1^ FW microgreen	[62]
Selenium (Se)	0.5 μg∙100 g^−1^ FW	[103]
	1.95–296.4 µg∙g^−1^ DW microgreens	[105]
Manganese (Mn)	0.159 mg∙100 g^−1^ FW	[103]
	9.99–22.9 µg∙g^−1^ FW	[70]
	0.29 mg∙100 g^−1^ FW microgreens	[62]
Natrium (Na)	65 mg∙100 g^−1^ FW	[103]
	194.33–1056 µg∙g^−1^ FW	[70]
	42 mg∙100 g^−1^ FW microgreen	[62]
Borum (B)	1.103–2.296 µg∙g^−1^ FW	[70]

Mineral composition of *B. rapa* subsp. *chinensis* is characterized by relatively high levels of potassium, calcium, and iron. Potassium constitutes a major fraction, accounting for 52–62% of total mineral content, with concentrations ranging from 8683 to 12,113 µg∙g^−1^ FW [70]. Calcium content reaches approximately 105 mg∙100 g^−1^ FW, while iron and magnesium occur at 0.8 mg∙100 g^−1^ FW and 19 mg∙100 g^−1^ FW, respectively (Table 12) [103].

Differences between developmental stages are evident, with microgreens exhibiting lower calcium (58 mg∙100 g^−1^ FW) and iron (0.49 mg∙100 g^−1^ FW) but higher levels of potassium (284 mg∙100 g^−1^ FW), magnesium (31 mg∙100 g^−1^ FW), zinc (0.30 mg∙100 g^−1^ FW), and manganese (0.29 mg∙100 g^−1^ FW) compared with mature leaves [62]. Additionally, *B. rapa* subsp. *chinensis* demonstrates a notable capacity for selenium accumulation, making it a potential dietary source for addressing selenium deficiencies [105,106]. Among trace elements, copper occurs at the lowest concentrations, ranging from 0.219 to 0.979 µg∙g^−1^ FW [70].

The composition of vitamins and minerals in *B. rapa* subsp. *chinensis* exhibits significant variability, influenced primarily by genetic factors, as well as environmental conditions such as climate, soil composition, irrigation, and cultivation practices [7,8]. Controlled cultivation systems, including hydroponics and LED-assisted growth, have been shown to affect metabolite profiles, including vitamin and pigment content [96,98]. In particular, nitrogen fertilization promotes biomass accumulation and influences nutrient composition, while abiotic stress factors may enhance the synthesis of bioactive compounds, including vitamins and secondary metabolites [5,107].

Differences in the distribution of nutrients among plant organs further contribute to compositional heterogeneity, with leaves, microgreens, roots, and seeds representing distinct reservoirs of bioactive compounds [19]. Post-harvest handling and storage conditions additionally affect nutrient stability and availability [7].

The complexity of plant metabolite composition implies that the biological activity of *B. rapa* subsp. *chinensis* results from synergistic interactions among multiple compounds rather than the effect of individual constituents. While certain components may enhance each other’s activity, others may interfere with metabolic pathways, influencing the overall health impact of the plant [108].

From an applied perspective, understanding the variability in chemical composition supports the selection of cultivars with desired nutritional profiles and contributes to the development of high-value crops for both agriculture and human nutrition [16]. Given that a substantial proportion of bioactive compounds used in medicine are of natural origin, plant-derived metabolites continue to represent a major area of interest for both the food and pharmaceutical sectors [109].

## 7. Health-Promoting Properties

The relationship between diet and human health is increasingly recognized, with growing emphasis on plant-based foods as sources of bioactive compounds supporting physiological functions [110]. In this context, *B. rapa* subsp. *chinensis*, as a representative of the *Brassicaceae* family, is considered a valuable dietary component with potential health-promoting properties, susceptible to preventing many diseases [5,19].

Contemporary therapeutic approaches to chronic diseases are often based on intensive pharmacotherapy, which may lead to polypharmacy and associated adverse effects, particularly in elderly and oncological patients [49,111]. This has increased interest in nutritional strategies based on dietary modification and the regular consumption of bioactive-rich plant foods [112].

Different forms of *B. rapa* subsp. *chinensis* can be selected to suit the flavor and health benefits. The main health-related properties and their associated chemical constituents are summarized in Table 13.

Furthermore, different plant organs (leaves, microgreens, roots, seeds) represent distinct reservoirs of bioactive compounds, contributing to compositional diversity within the species [19]. Post-harvest handling and storage conditions also play a crucial role in maintaining nutritional quality [7]. Understanding this variability is essential for optimizing cultivation strategies and selecting cultivars with desirable nutritional profiles. Such knowledge supports the development of high-value plant materials tailored for dietary and therapeutic applications [16].

The way of taking natural substances by humans, initiated by biting, grinding, and chewing, activates enzymes and thus the organs of the digestive system to work, triggering a whole cascade of complex changes in the body that are fundamental to overall health [112,113].

**Table 13:** Health-related effects and functional applications of *B. rapa* subsp. *chinensis* after short-term heat treatment.

Functioning	Application	Chemical Constituent Responsible for Functioning	References
detoxification	liver failure	glucosinolates isothiocyanates polyphenols fiber (cellulose, pectins) plant pigments	[114,115]
	renal dysfunction		[63]
	Coprostasis		[49]
	taking xenobiotics (smoking, medications)		[116]
antioxidant activity	improper diet	polyphenols glucosinolates vitamins minerals	[16]
	stress and fatigue		[114]
	skin aging		[103]
	inflammation of the digestive system		[49]
anti-inflammatory	inflammation of the digestive system organs	glucosinolates isothiocyanates polyphenols carotenoids	[23]
	hepatitis b		[114]
	polyps in the digestive system		[117]
	Pneumonia		[94,112]
cancer-preventive potential	oral cancer	isothiocyanates nitriles glucosinolates polyphenols flavonoids plant pigments	[49]
	hepatocellular carcinoma		[18]
	stomach cancer		[103]
	pancreatic cancer		[19]
	intestinal cancer		[103]
	prostate cancer		[117]
	breast cancer		[20]
	vulvar cancer		[118]
	ovarian/uterine/vaginal cancer		[118]
	chemotherapy/radiotherapy		[29]
prebiotic effect	obstructions	isothiocyanates glucosinolates fiber	[3]
	slowing down of intestinal peristalsis		[16]
	leaky gut syndrome		[3]
slimming	Overweight	plants pigments fiber glucosinolates water content	[116]
	abdominal obesity		[20]
	Cellulitis		[49]
antiatherogenic effect	Obesity	polyphenols (anthocyanins) isothiocyanates glucosinolates	[119]
	cirrhosis of the liver		[120]
	hypertension		[94]
antidiabetic	type 2 diabetes	fiber polyphenols glucosinolates isothiocyanates	[61]
	insulin resistance		[121]
strengthening	frequent infections	flavonoles isothiocyanates vitamins and minerals	[28]
	osteopenia/osteoporosis		[29]
	micronutrient and vitamin deficiencies		[103]

### 7.1. Antioxidant Effects—Scavenging of Free Radicals

#### Biochemical and In Vitro Experiments

The antioxidant system of *Brassicaceae* plants is based on the coordinated activity of enzymatic defense mechanisms responsible for neutralizing reactive oxygen species (ROS) generated under stress conditions [122]. Key enzymes include superoxide dismutase (SOD), which catalyzes the conversion of superoxide radicals into oxygen and hydrogen peroxide, catalase (CAT), which decomposes hydrogen peroxide into water and oxygen, and peroxidases (POX/POD), which further participate in hydrogen peroxide detoxification and plant defense responses [73,123].

In *B. rapa* subsp. *chinensis*, high enzymatic antioxidant activity has been reported. The cultivar ‘Pak choi White’ exhibited catalase activity at 44.96 mM H_2_O_2_/g FW/min and peroxidase activity at 34.85 ΔA/min/g FW, exceeding values reported for other cruciferous vegetables such as kale. The unit ΔA quantifies the dynamic rate of defense enzyme activity by measuring the rate at which one gram of fresh plant tissue changes the color of a test solution per minute. It expresses the optical change in fluid density (absorbance), which provides direct evidence of the plant’s biochemical effectiveness in neutralizing harmful hydrogen peroxide [73]. In contrast, Tabassam et al. reported lower activity ranges of 6–9 U/g FW for catalase and 4.8–8.5 U/g FW for peroxidase, indicating variability depending on experimental conditions and plant material. This standardized unit of enzymatic activity defines the catalytic potential of proteins contained in one gram of fresh tissue weight, where one unit (U) is defined by a specific biochemical effect, such as, for example, fifty percent inhibition of a photochemical reaction in the case of superoxide dismutase (SOD) assays [123].

Beyond enzymatic systems, antioxidant activity in *B. rapa* subsp. *chinensis* is strongly influenced by secondary metabolites. These compounds extend their role beyond plant defense and contribute significantly to biological effects in humans [124]. Polyphenols, especially flavonoids such as kaempferol, quercetin, and isorhamnetin, are major contributors to antioxidant activity, acting as effective scavengers of free radicals [16,31,103]. Glycosylated derivatives, including kaempferol-3,7-di-O-glucoside and related compounds, further enhance this activity [9].

Glucosinolates and their hydrolysis products, particularly isothiocyanates, also participate in antioxidant and cytoprotective mechanisms. These compounds may stabilize DNA and inhibit oxidative damage, while some additionally exhibit antimicrobial activity, including inhibition of *Escherichia coli* growth [63,125,126]. Sulforaphane, one of the key isothiocyanates, activates the Nrf2 signaling pathway, enhancing cellular defense against oxidative stress.

Experimental studies confirm the biological relevance of these effects. Extracts of *B. rapa* subsp. *chinensis* reduced reactive oxygen species levels in macrophages by 28.2% at a concentration of 500 μg/mL, exceeding standard control groups [28]. Additionally, cultivar-dependent differences have been observed, with green and light-green cultivars exhibiting DPPH radical scavenging activity of 21.07% and 18.39%, respectively, at the highest tested concentrations [92].

Quantitative analyses using different methods further illustrate the antioxidant potential of *B. rapa* subsp. *chinensis*. Radical scavenging activity (RSA) reached 29.98%, while the Trolox equivalent antioxidant capacity (TEAC) was 27.35 mM in plant extracts dominated by phenolic acids [127]. Ferric reducing antioxidant power (FRAP) values ranged from 5 to 7 µmol/g FW and showed a strong positive correlation with polyphenol, flavonoid, and vitamin C content. In parallel, DPPH radical scavenging activity remained at approximately 97% in some experimental systems, highlighting the strong antioxidant capacity of the plant [124].

Non-phenolic compounds, including carotenoids and chlorophylls, also contribute to antioxidant capacity. These isoprenoid pigments interact with lipid components of cell membranes, protecting them against oxidative damage caused by internal and external factors such as UV radiation [91,94]. Carotenoids additionally serve as precursors of vitamin A and play a role in electron transport and cellular energy metabolism [128].

Variability in antioxidant activity is associated with differences in enzymatic profiles, metabolite composition, and environmental conditions. The overall scavenging capacity of *B. rapa* subsp. *chinensis* results from the combined action of multiple compounds, including vitamins, minerals, amino acids, and polysaccharides, accumulated under specific stress and cultivation conditions [129].

From a physiological perspective, imbalance in oxidative status is associated with the development of cardiovascular, immune, and neurodegenerative disorders and is closely linked with inflammatory processes [5]. Accordingly, the antioxidant activity of *B. rapa* subsp. *chinensis* is strongly correlated with its anti-inflammatory potential, highlighting its relevance in preventive nutrition and health maintenance [28].

### 7.2. Anti-Inflammatory Activity

Inflammatory processes, particularly within the gastrointestinal tract and other organ systems, are closely associated with immune activation and increased production of pro-inflammatory cytokines, including interleukin-6 (IL-6), interleukin-1β (IL-1β) and tumor necrosis factor α (TNF-α). Prolonged and uncontrolled inflammation may promote carcinogenesis in various tissues [130]. Bioactive compounds present in *B. rapa* subsp. *chinensis* contribute to the modulation of these pathways. Polyphenols, including phenolic acids and flavonoids, have been shown to reduce levels of IL-6 and IL-1β, indicating their role in immunomodulation and anti-inflammatory activity [131]. Flavonols such as kaempferol, quercetin, and isorhamnetin additionally exhibit antimicrobial activity, inhibiting the growth of bacteria, fungi, and viruses [9].

#### 7.2.1. In Vitro Experiments—Cell Lines

Phenolic acids such as p-coumaric, chlorogenic, ferulic, and caffeic acids, along with quercetin, have been shown to reduce excessive leukocyte adhesion to vascular endothelium, a process stimulated by TNF-α, indicating the anti-inflammatory potential of these compounds [132]. In addition, compounds such as syringin exhibit hepatoprotective properties and contribute to the reduction in inflammatory processes in liver tissues [16]. Aqueous extracts of plant material *B. rapa* subsp. *chinensis* (50–100 µg/mL) demonstrated reduced levels of IL-6, IL-1β, TNF-α, inducible nitric oxide synthase (iNOS), and nitric oxide (NO) in RAW 264.7 macrophages, alongside modulation of TLR2/NF-κB, JNK, and Akt signaling pathways [132].

#### 7.2.2. In Vivo Experiments

Experimental evidence indicates that combined exposure to phenolic compounds and glucosinolates may significantly reduce the size of non-neoplastic lesions in the colon by 53.9% and inhibit their progression by 31.6% [63]. The effect may be further enhanced by lifestyle factors such as physical activity, which has been associated with an increase in therapeutic response parameters.

Glucosinolates and their hydrolysis products, particularly isothiocyanates, play a central role in anti-inflammatory mechanisms. Sulforaphane has been shown to inhibit abnormal cell proliferation and exert anti-inflammatory effects through modulation of the Nrf2 signaling pathway and inhibition of NF-κB activity, including in cardiovascular and bone-related disorders [28,29,117].

Recent studies further support the importance of glucosinolate-derived compounds in regulating inflammation. Experiments in mice revealed that extracts of *B. rapa* subsp. *chinensis* enriched in glucosinolates, particularly phenethyl isothiocyanate and sulforaphane, exert anti-inflammatory effects via activation of the Nrf2 pathway in hepatocytes [133].

However, not all studies confirm direct modulation of NF-κB, as demonstrated in investigations of high-glucosinolate hybrids, where reductions in pro-inflammatory cytokine levels occurred independently of this pathway. These findings highlight the complexity of anti-inflammatory mechanisms in *B. rapa* subsp. *chinensis*, which may involve multiple overlapping signaling cascades depending on phytochemical composition [28].

The combined action of multiple compounds may lead to synergistic effects. For example, co-application of vitamin C with allicin resulted in enhanced inhibition of pro-inflammatory cytokine production in intestinal epithelial cells, involving pathways such as NF-κB, TLR4, and MyD88 [130]. These results illustrate the importance of considering interactions between dietary components rather than isolated compounds.

Overall, the anti-inflammatory activity of *B. rapa* subsp. *chinensis* is driven by the combined effects of polyphenols, glucosinolates, isothiocyanates, and vitamins, acting through multiple molecular pathways. This multifactorial mechanism positions the plant as a promising component of dietary strategies aimed at reducing inflammation and preventing inflammation-related diseases.

### 7.3. Anti-Cancer Potential

Epidemiological evidence suggests that regular consumption of cruciferous vegetables, including *B. rapa* subsp. *chinensis*, may reduce the risk of several cancers, particularly those associated with the gastrointestinal tract, urinary system, and hormone-dependent tissues, such as prostate and breast cancer [49,103]. These protective effects result from the combined action of vitamins, minerals, carotenoids, polyphenols, and especially glucosinolates and their bioactive derivatives [20,63].

Among the most intensively studied compounds are glucosinolates and their hydrolysis products, including isothiocyanates such as phenethyl isothiocyanate, benzyl isothiocyanate, erucin and sulforaphane, as well as indolic derivatives such as indole-3-carbinol (I3C) and N-methoxyindolyl-3-carbinol [34,103]. Their biological activity depends on enzymatic conversion and microbiota-mediated transformations in the gastrointestinal tract [63].

#### 7.3.1. In Vitro Experiments—Cell Lines

Phenethyl isothiocyanate (PEITC) represents another compound with well-documented anticancer properties. Its activity includes induction of apoptosis, inhibition of tumor cell proliferation, suppression of angiogenesis, and reduction in metastasis through multiple molecular mechanisms [134,135]. β-phenylethylisothiocyanate in vitro significantly suppresses the growth of prostate cancer cells (DU145 line) by precisely blocking the Janus Kinases JAK/ Signal Transducers and Activators of Transcription 3 STAT3 signaling pathway and significantly inhibits the proliferation of hepatocellular carcinoma cells (HepG2 line isolated from humans), which is determined in a dose-dependent manner [136].

Indole-3-carbinol (I3C) and its dimeric product diindolylmethane (DIM), formed under acidic conditions in the gastrointestinal tract, play an important role in the modulation of hormone-dependent cancers. These compounds activate the aryl hydrocarbon receptor (AhR), enhancing xenobiotic detoxification and influencing estrogen metabolism, thereby contributing to protective effects in breast, prostate, and gynecological cancers [115,118]. Additionally, they may enhance the effectiveness of modern oncological therapies, including immunotherapy approaches such as checkpoint inhibitors and CAR-T cell therapy [137].

#### 7.3.2. In Vivo Experiments

Sulforaphane, one of the most extensively studied isothiocyanates, has attracted considerable attention due to its strong chemopreventive potential. It modulates key cellular pathways, including activation of phase II detoxification enzymes, regulation of oxidative stress via the Nrf2 pathway, and epigenetic modulation through inhibition of histone deacetylases (HDAC) [29,117]. Experimental studies have demonstrated its ability to suppress tumor growth in animal models, reducing prostate tumor size by over 50% after short-term treatment and up to approximately 70% with prolonged administration [138]. Similar effects have been reported in breast cancer models, with tumor reduction ranging from 20% to 60% depending on dose and treatment duration [139].

In human studies, sulforaphane has shown potential in reducing cancer-associated molecular markers, particularly when administered at doses of ≥200 µmol/day or ≥200 µmol glucoraphanin per week over prolonged periods. Notably, the most significant effects were observed in prostate cancer, although changes in clinical biomarkers such as prostate-specific antigen (PSA) were not consistently detected [117]. Furthermore, sulforaphane has been reported to enhance the efficacy of conventional chemotherapy by sensitizing cancer cells while reducing adverse side effects, indicating its potential as an adjunct therapeutic agent [29].

Other glucosinolate-derived compounds, including nitriles such as 3-phenylpropionitrile, may also contribute to anticancer activity through interactions with isothiocyanates, although their role requires further investigation [3].

Despite promising results, the biological effects of these compounds are influenced by their bioavailability, metabolism and rapid excretion. Isothiocyanates are largely eliminated via the kidneys within approximately 48 h, which complicates the design of long-term dietary intervention studies [23,140]. To overcome these limitations, novel delivery strategies, including nanoformulations, are being developed to improve stability and bioavailability of these bioactive compounds [139].

Importantly, the anticancer potential of *B. rapa* subsp. *chinensis* is not limited to glucosinolates. Flavonoids and phenolic acids also contribute to cytoprotective effects, including inhibition of cancer cell proliferation and reduction in oxidative damage at early stages of carcinogenesis [59,63]. Overall, the anticancer activity of *B. rapa* subsp. *chinensis* results from a complex interplay of multiple phytochemicals acting through diverse molecular pathways. There is a lack of additional data from standardized extract applications, that would provide insight into the anti-cancer application of this plant. Although a dozen experimental studies demonstrate significant potential, further clinical investigations are required to establish optimal doses, bioavailability, and long-term effects in humans.

### 7.4. Metabolic Effects—Impact on Lipid and Glucose Metabolism

Metabolic syndrome comprises a cluster of interconnected disorders, including abdominal obesity, hypertension, insulin resistance and hepatic dysfunction, which collectively contribute to the development of cardiovascular disease, atherosclerosis and type 2 diabetes [49]. Lifestyle factors, particularly diet and physical activity, play a central role in both the development and prevention of these conditions [18].

Dietary strategies emphasizing plant-based foods have been associated with improved metabolic outcomes, including reduced triglyceride levels and improved lipid profiles [141]. In this context, *B. rapa* subsp. *chinensis* represents a low-calorie, water-rich vegetable containing dietary fiber and bioactive compounds that support metabolic regulation [61]. The combination of high water content and fiber contributes to increased satiety, improved gastrointestinal motility, and better appetite control, which is particularly beneficial in individuals with obesity, insulin resistance and type 2 diabetes [121].

Bioactive phytochemicals present in *B. rapa* subsp. *chinensis*, including polyphenols and isothiocyanates, contribute to the regulation of lipid and glucose metabolism [20,142]. Flavonoid derivatives, such as isorhamnetin glycosides, have been reported to influence endocrine activity by modulating secretion of peptide hormones associated with glucose homeostasis [9]. Additionally, compounds characteristic of *Brassicaceae* plants have been shown to increase tissue sensitivity to insulin, supporting glycemic control [3,26,143].

Beyond direct metabolic effects, *B. rapa* subsp. *chinensis* provides essential micronutrients and vitamins involved in metabolic processes, including coenzyme A synthesis and enzymatic regulation [49]. Leafy vegetables, including this species, have also been associated with maintaining bone mineralization, which may indirectly support metabolic homeostasis [144].

#### 7.4.1. In Vitro Experiments

In addition to systemic metabolic effects, extracts of *B. rapa* subsp. *chinensis* have demonstrated inhibitory activity against digestive enzymes. In vitro studies revealed inhibition of α-amylase and α-glucosidase, key enzymes involved in carbohydrate digestion, suggesting a mechanism for improved glycemic control [143]. Furthermore, dichloromethane extracts from leaves inhibited pancreatic lipase activity with an IC_50_ value of 352.1 µg·mL^−1^, indicating potential for regulating lipid absorption and supporting weight management [145].

#### 7.4.2. In Vivo Experiments

Experimental studies provide further insight into the molecular mechanisms involved. Glucoraphanin-rich extracts have been shown to reduce low-density lipoprotein (LDL) cholesterol levels and enhance oxidative phosphorylation efficiency [117]. Phenethyl isothiocyanate administration (30–75 mg/kg/day) in C57BL/6 rodent models improved liver function and reduced lipid accumulation in hepatic tissues under high-fat diet conditions [146].

Similarly, supplementation with freeze-dried *B. rapa* subsp. *chinensis* leaves (5–7% of diet) modulated the expression of genes associated with lipid metabolism in Syrian golden hamsters over a 56-day period. This effect involved activation of the PPAR-α signaling pathway, increased cytochrome P450 27A1 (CYP27A1) activity responsible for cholesterol metabolism, and reduction of 3-hydroxy-3-methylglutaryl-CoA (HMG-CoA) reductase activity, a key enzyme in cholesterol biosynthesis [141].

Overall, the metabolic effects of *B. rapa* subsp. *chinensis* result from the combined action of bioactive phytochemicals, which act synergistically to regulate lipid metabolism, improve glucose homeostasis, and support liver function. Consequently, regular consumption of this plant may contribute to the prevention and management of metabolic disorders, particularly when combined with appropriate lifestyle interventions.

### 7.5. Gut Microbiota Modulation—Prebiotic Effects

*Brassicaceae* vegetables, including *B. rapa* L. subsp. *chinensis*, represent an important dietary component supporting gut microbiota modulation. Prebiotic effects of *B. rapa* subsp. *chinensis* are primarily related to its content of dietary fiber and similar bioactive compounds, including sugar alcohols and complex polysaccharides. These components stimulate microbial activity in the lower gastrointestinal tract, enhancing bacterial fermentation processes and promoting the growth of beneficial microbial populations [3]. In addition, seeds and other plant parts may serve as concentrated sources of indigestible carbohydrates and antioxidants, further supporting microbial diversity [63,147].

#### 7.5.1. In Vitro Experiments

In vitro fermentation studies demonstrated that pak choi microgreens increased the production of SCFAs to 4.64 mmol/g DW, along with a higher abundance of bacterial taxa such as *Monoglobus* and *Erysipelotrichaceae* UCG-003 [148]. These metabolites, particularly butyrate, acetate, and propionate, play a key role in maintaining intestinal barrier integrity and regulating host metabolism.

The modulation of gut microbiota by *Brassicaceae* vegetables is also influenced by specific phytochemicals. Anthocyanins present in purple cultivars may stimulate the growth of beneficial genera such as *Bifidobacterium* and *Lactobacillus*, while suppressing pathogenic microorganisms including *Escherichia coli* and *Salmonella* spp. [119]. In parallel, glucosinolate-derived compounds have been associated with an increased abundance of Gram-positive bacteria, particularly lactobacilli, contributing to improved gut health [149].

#### 7.5.2. In Vivo Experiments

Experimental studies provide evidence for microbiota modulation by *B. rapa* subsp. *chinensis*. In a murine model of colitis (DSS-induced), combined administration of vitamin C and allicin from plant extracts resulted in a significant improvement in gut microbial composition, including increased abundance of short-chain fatty acid (SCFA)-producing bacteria such as *Allobaculum*, *Odoribacter*, *Verrucomicrobia*, and *Parabacteroides*. These changes were associated with reduced inflammation and improved intestinal epithelial function in tested animals [130].

Glucoraphanin, a glucosinolate present in *B. rapa* subsp. *chinensis*, has been shown to protect intestinal tissues against chronic inflammation and to support the integrity of the intestinal barrier [150]. These effects further emphasize the role of glucosinolate metabolism in host–microbiota interactions.

Importantly, the bioavailability and biological activity of phytochemicals are strongly influenced by microbial metabolism. The large intestine represents a key site for transformation of polyphenols and flavonoids, where microbial enzymes enhance their biological activity and absorption [5]. Moreover, interactions between gut microbiota and glucosinolate metabolism determine the efficiency of isothiocyanate formation, even in the absence of plant-derived myrosinase [151].

Overall, the interaction between *B. rapa* subsp. *chinensis* and gut microbiota reflects a bidirectional relationship, in which dietary phytochemicals modulate microbial composition, while microbiota influences the bioavailability and activity of plant-derived compounds. This complex interplay underlies the prebiotic potential of this species and supports its role in maintaining gastrointestinal and systemic health.

## 8. Health Risks—Negative Side

While numerous studies emphasize the health benefits of *Brassicaceae* vegetables, including *B. rapa* L. subsp. *chinensis*, potential risks associated with their consumption should also be critically considered [23]. A comprehensive evaluation of both beneficial and adverse effects is essential when assessing their role in human nutrition, particularly in the context of large-scale dietary recommendations and food production systems [17].

*Brassicaceae* species, particular attention is given to glucosinolates and their degradation products. Although these compounds are widely recognized for their beneficial biological properties, excessive intake or imbalanced metabolism may lead to adverse effects, including goitrogenic activity or disturbances in thyroid function, especially under conditions of iodine deficiency. This aspect highlights the importance of dose and dietary context when evaluating their effects [152,153].

Anti-nutritional factors, such as oxalates and phytates, may also influence mineral bioavailability by forming complexes with calcium, iron, and other essential elements. This may reduce their absorption and nutritional value, particularly in diets already deficient in these micronutrients [154].

Another important consideration is the presence of nitrates in leafy vegetables, including *B. rapa* subsp. *chinensis*, which may accumulate depending on nitrogen fertilization and environmental conditions. High nitrate intake can be associated with potential health risks, particularly in sensitive populations, due to conversion into nitrites and subsequent formation of potentially harmful compounds [155,156].

Processing and preparation methods further affect both beneficial and adverse properties. Improper storage or prolonged processing may result in degradation of bioactive compounds or formation of undesirable metabolites. Conversely, certain culinary processes may reduce the content of potentially harmful components, demonstrating the importance of appropriate food preparation [7].

Individual variability represents an additional limitation in evaluating the safety profile of *B. rapa* subsp. *chinensis*. Differences in gut microbiota composition, metabolic capacity, age, and health status may influence the transformation and bioactivity of plant-derived compounds, leading to variable physiological responses among individuals [5,120,150,157].

Overall, the potential risks associated with the consumption of *B. rapa* subsp. *chinensis* are closely related to dosage, cultivation conditions, and individual susceptibility. While the overall benefit–risk balance remains strongly favorable, these factors should be considered in both nutritional recommendations and future research [13,158,159].

### 8.1. Antinutritional Factors

The term “antinutritional factors” refers to naturally occurring plant compounds that may interfere with nutrient utilization or exert adverse physiological effects under specific conditions [20]. In *Brassicaceae* vegetables, including *B. rapa* subsp. *chinensis*, these compounds include protease inhibitors, certain flavonoids, and non-protein amino acid derivatives. Among them, goitrogenic substances associated with glucosinolate metabolism represent a particularly important group.

Goitrogens, including thiocyanates, isothiocyanates, and related sulfur-containing compounds, may affect the function of the thyroid gland by interfering with hormone synthesis and iodine metabolism [153,160]. Selected glucosinolates such as progoitrin, gluconapin, neoglucobrassicin, and 4-methoxyglucobrassicin have been identified as potential contributors to these effects [161]. These compounds are generally hydrophilic, which limits their ability to penetrate biological membranes and form stable DNA adducts; therefore, adverse effects are typically associated with high doses or prolonged exposure [162].

The biological activity of glucosinolate derivatives is highly dose-dependent. While moderate intake is considered safe, excessive consumption or combined exposure to multiple goitrogenic factors may increase the risk of thyroid dysfunction, particularly under conditions of iodine deficiency or additional environmental exposure to elements such as lithium [160]. A daily intake of approximately 100–200 g of cruciferous vegetables is generally regarded as safe and does not result in significant adverse effects [163].

**Figure 7:**
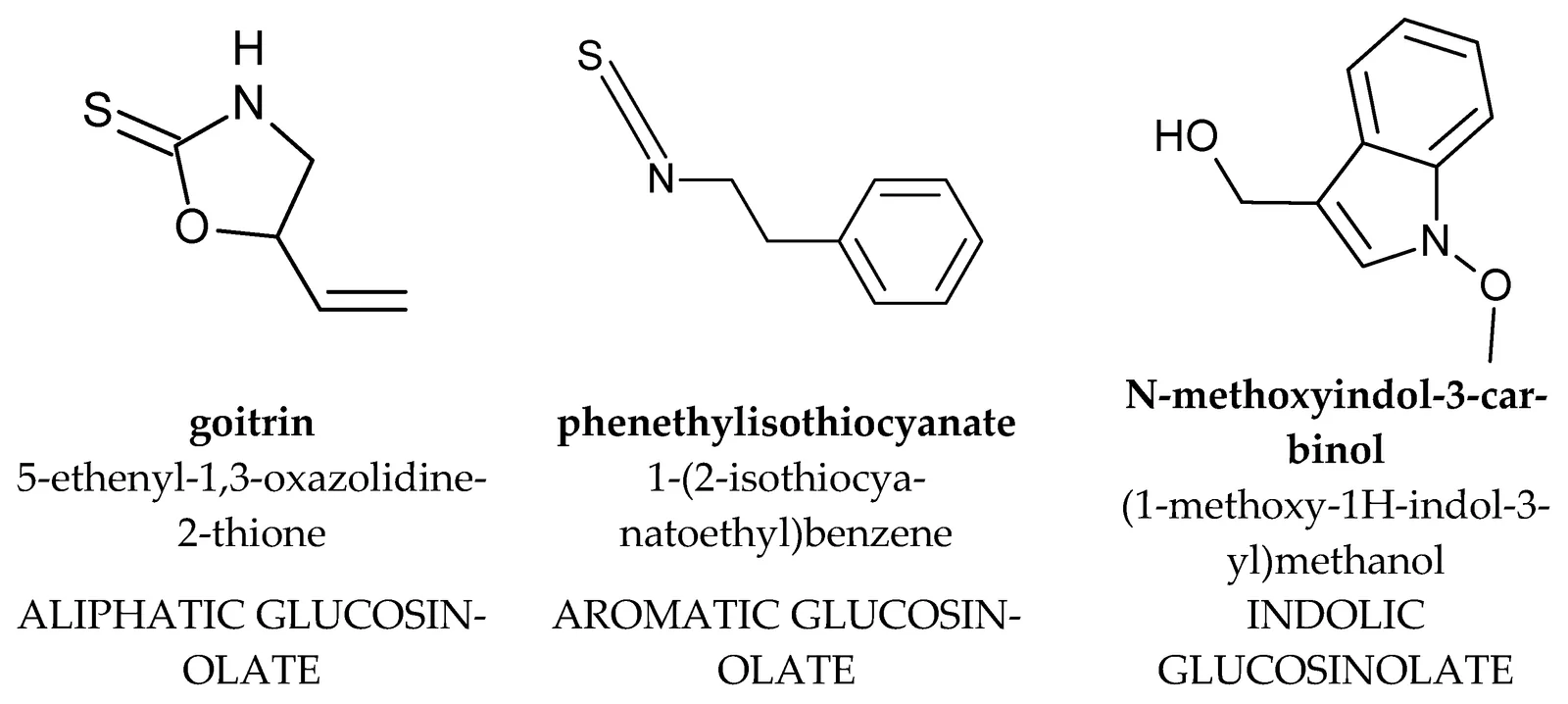
Selected glucosinolate-derived compounds with potential adverse biological effects identified in *B. rapa* subsp. *chinensis*.

Smaller isothiocyanate molecules, in turn, are characterized by lipophilicity, which facilitates the formation of bonds with proteins or nucleic acids. Negative biological effects are primarily attributed to derivatives of tryptophan or aliphatic amino acids, which are more active than the source compounds—indole isothiocyanates and aliphatic amino acids [162] (Figure 7).

Individual susceptibility represents an important factor influencing the safety profile of *B. rapa* subsp. *chinensis*. In particular, individuals with a predisposition to thyroid disorders may be more sensitive to the effects of goitrogenic compounds. In such cases, excessive consumption of raw cruciferous vegetables may lead to reduced thyroxine (T4) levels and compensatory enlargement of the thyroid gland [152].

Importantly, appropriate food processing methods may significantly reduce the content of antinutritional compounds. Thermal treatment, fermentation, and controlled preparation practices can decrease glucosinolate content and limit the formation of potentially harmful metabolites, thereby improving the safety of consumption while preserving nutritional benefits [13].

Overall, the potential adverse effects associated with antinutritional factors in *B. rapa* subsp. *chinensis* are primarily dependent on dose, dietary context, and individual susceptibility [164]. When consumed as part of a balanced diet and prepared appropriately, the health benefits of this species strongly outweigh potential risks [152,165].

### 8.2. Contaminants

*B. rapa* subsp. *chinensis* is recognized for its high capacity to absorb mineral elements from the soil, including beneficial micronutrients such as selenium (Se), which contributes to its nutritional value [106]. However, this property also results in the potential accumulation of undesirable contaminants, particularly when plants are cultivated in polluted environments.

As a representative of leafy vegetables, *B. rapa* subsp. *chinensis* exhibits a relatively high capacity for uptake of heavy metals and other xenobiotics from soil and water. Reported contaminants include copper (Cu), cadmium (Cd), chromium (Cr), arsenic (As), cerium (Ce), lanthanum (La), and fluorine (F), which may accumulate in plant tissues depending on environmental conditions [123,166]. Among these, arsenic has been analytically confirmed to penetrate edible tissues of this species [123].

Cultivation in contaminated soils may lead to significant metabolic alterations in plant tissues, including a reduction in the content of bioactive compounds and overall biological activity. At the same time, consumption of contaminated plant material increases the risk of toxic exposure in humans [167]. Bioavailability of heavy metals varies widely; for example, chromium (Cr), nickel (Ni), arsenic (As), and copper (Cu) may be absorbed in the gastrointestinal tract at levels ranging from 6.92% to 81.15% [168].

Quantitative assessments indicate that dietary intake from pak choi may contribute up to 0.01 mg/kg/day of cadmium (Cd) and 0.22 mg/kg/day of chromium (Cr), values that may exceed permissible intake levels under certain conditions [169]. Lead (Pb) concentrations in plant material have been reported in the range of 0.310–0.512 µg/g, exceeding regulatory limits established for leafy vegetables (0.3 mg/kg) in European legislation [70].

The accumulation of toxic elements is associated with potential bioaccumulation in human organs, particularly the liver and kidneys. Even low concentrations of certain heavy metals may disrupt physiological homeostasis. Additional environmental contaminants, such as radionuclides (e.g., ^210^Po), have also been detected in vegetable tissues at average levels of 0.7 Bq/kg, further emphasizing the need for monitoring food safety [170].

Agricultural practices represent another significant source of contamination. Intensive fertilization, particularly with nitrogen-based fertilizers, promotes plant growth but may lead to excessive accumulation of nitrates (NO_3_^−^) in leaves and stems [155]. These compounds may negatively affect both plant metabolism and human health. In plants, excess nitrates disrupt photosynthetic processes and reduce the content of bioactive compounds, including vitamins and isoprenoids [156]. In humans, nitrates and their derivatives are classified as potentially carcinogenic due to their conversion into nitrites and N-nitroso compounds [171].

Emerging contaminants (CECs), including pharmaceutical residues, represent an additional and insufficiently explored category of pollutants in plant-derived foods. Their presence may result from environmental contamination and wastewater reuse, raising concerns regarding long-term exposure and health effects [10].

Pesticides constitute another important group of xenobiotics associated with pak choi cultivation. Studies indicate that a significant proportion of market samples may contain detectable residues. For example, 77.27% of analyzed samples contained pesticide residues, including pyridaben, carbendazim, and acetamiprid, with some compounds not approved for use [172]. Chronic exposure to pesticide residues has been associated with neurotoxicity, endocrine disruption, and mutagenesis [172,173].

Microbiological contamination also presents a relevant safety concern, particularly in the case of microgreens. Cultivation conditions, including high humidity and stagnant water, favor the growth of pathogenic microorganisms. Roots and sprouts are especially susceptible to contamination due to direct contact with soil and nutrient solutions [20,174]. Reported microorganisms include *Salmonella enterica*, *Escherichia coli*, *Klebsiella variicola*, *Klebsiella pneumoniae*, and *Citrobacter freundii*, with Total counts of Enterobacteriaceae exceeding 5.00 log CFU/g in some cases [174,175].

Preventive strategies include appropriate hygienic practices, such as seed disinfection using calcium hypochlorite and lactic acid solutions, controlled storage conditions, low-temperature storage, and modified-atmosphere packaging. These measures can reduce the microbial load but may not completely eliminate contamination [176,177].

Ensuring food safety requires strict regulation of cultivation and distribution processes, particularly in relation to the use of fertilizers, pesticides, and other agrochemicals. The FAO emphasizes the importance of sustainable and environmentally friendly agricultural practices, including organic farming, to minimize contamination risks [20].

In recent years, innovative approaches such as nanotechnology have been explored to reduce uptake of toxic substances and improve plant quality. The application of nanoparticles has been shown to enhance physicochemical and biological properties of the cultivation substrate, supporting plant growth while limiting contaminant accumulation [178].

Overall, the safety of *B. rapa* subsp. *chinensis* depends strongly on environmental conditions, cultivation practices, and post-harvest handling. While the species possesses high nutritional value, effective risk management is essential to minimize exposure to contaminants and ensure its suitability as a functional food.

### 8.3. General Dietary Contraindications

Although *B. rapa* subsp. *chinensis* is widely recognized for its nutritional and health-promoting properties, its consumption may not be equally beneficial for all population groups (Table 14). Identification of dietary contraindications is essential for safe nutritional recommendations and personalized diet planning.

One of the primary concerns relates to thyroid disorders. In individuals with hypothyroidism, particularly Hashimoto’s disease, glucosinolate-derived goitrogenic compounds may interfere with iodine uptake and thyroid hormone synthesis. Although moderate consumption of cruciferous vegetables is generally considered safe, excessive intake—especially in raw form—may lead to clinically relevant disturbances. For example, a case study described severe hypothyroidism associated with the consumption of approximately 1500 g of fresh pak choi daily, resulting in elevated TSH levels (74.4 mIU/L) and suppression of thyroid function [158]. Thermal processing and adequate iodine intake may mitigate these effects [154,179].

Mechanical properties of the plant tissue may also limit its suitability for certain individuals. Due to the relatively firm texture of leaf petioles, consumption of raw pak choi may pose difficulties for patients with dysphagia, impaired swallowing reflex, or poor oral health, particularly in elderly populations, where such conditions affect up to 42% of individuals over 60 years of age [111,180]. Modification of texture through cooking or processing significantly improves digestibility and safety.

Gastrointestinal conditions represent another important contraindication. In individuals with acute digestive disorders, hypersensitivity, or inflammatory bowel diseases such as Crohn’s disease, consumption of raw cruciferous vegetables may exacerbate symptoms, including bloating, diarrhea, and mucosal irritation. In such cases, intake should be limited or restricted to thermally processed forms and introduced gradually [23,140,149].

Drug–nutrient interactions are also relevant. Vitamin K present in *B. rapa* subsp. *chinensis* may interact with anticoagulant medications, potentially reducing their therapeutic efficacy. Therefore, controlled intake and temporal separation between medication use and consumption are recommended [140].

Culinary practices may further influence both safety and nutritional quality. High-temperature processing, particularly frying in very hot oil, may lead to degradation of beneficial compounds and formation of undesirable products, thus reducing the overall health value of the vegetable [13].

Finally, the variability of plant composition represents a challenge in defining universal intake recommendations. Differences in cultivar, cultivation conditions and individual metabolic responses result in heterogeneous effects, making precise standardization of dosage and physiological outcomes difficult [158]. Overall, while *B. rapa* subsp. *chinensis* is generally safe and beneficial for most individuals, its consumption should be tailored to individual health status, dietary habits, and potential medical conditions.

## 9. Dietary, Pharmaceutical, and Cosmetic Applications

### 9.1. Potential in Diets, Dietary Supplements, and Nutraceuticals

*B. rapa* subsp. *chinensis*, volatile compounds such as esters and terpenes contribute to a distinctive sensory profile, with considerable variability among cultivars. For example, the Xiangqingcai variety has been described as having aroma characteristics similar to cooked rice (*Oryza sativa*), illustrating the influence of genetic diversity on flavor perception and consumer acceptance [181].

The culinary applications of *B. rapa* subsp. *chinensis* are diverse and continuously expanding. The plant may be consumed in raw forms, such as juices and smoothies, or incorporated into processed products, including flours, bread, or bakery items enriched with microgreens or leaf purees [182,183,184]. Traditional uses also exist, such as drying and powdering leaves for later utilization in local cuisines, demonstrating its adaptability across cultures [185]. In addition, plant-derived pigments, including chlorophylls and anthocyanins, are increasingly used as natural colorants in food products [31,94].

Beyond direct consumption, *B. rapa* subsp. *chinensis* represents a promising source of bioactive compounds for food preservation and functional ingredient development. Extracts rich in antioxidants and antimicrobial compounds have been investigated as natural alternatives to synthetic preservatives. For example, fermented (e.g., kimchi-derived products) and freeze-dried preparations demonstrated the ability to maintain color stability and reduce lipid oxidation in meat products, highlighting their potential in improving food quality [25].

Antimicrobial activity of *B. rapa* subsp. *chinensis* extracts has also been demonstrated in vitro. Ethanolic extracts inhibited the growth of pathogenic bacteria such as *Salmonella enteritidis*, although with lower efficacy compared to standard antibiotics such as ciprofloxacin. Reduced activity was observed against *Escherichia coli* and *Staphylococcus aureus*, indicating moderate and selective antimicrobial potential [186]. Similar findings were reported in disk diffusion assays, where inhibition zones (7–10 mm) were significantly smaller than those observed for antibiotic controls [187]. These results suggest that plant extracts may serve as complementary rather than primary antimicrobial agents.

In addition to antimicrobial effects, bioactive compounds derived from *B. rapa* subsp. *chinensis* have shown potential in modulating pathogenic mechanisms, such as inhibition of lipase activity in *Acinetobacter baumannii*, a bacterium associated with opportunistic infections [82]. Such findings highlight the potential for developing plant-based antimicrobial strategies targeting specific bacterial pathways.

From a clinical nutrition perspective, *B. rapa* subsp. *chinensis* may serve as a component of dietary interventions supporting patients with chronic diseases. Regular consumption of plant-derived foods is associated with reduced side effects compared to pharmacotherapy and may provide protective effects on the gastrointestinal system [188,189]. The plant may therefore support nutritional strategies complementing conventional therapy.

Preservation of bioactive compounds remains a key challenge due to the high water content and susceptibility of tissues to degradation. Processing into extracts or stabilized formulations may enhance shelf life and biological activity [88]. In particular, improving the stability and bioavailability of compounds such as sulforaphane represents an important research direction. Nanotechnology-based delivery systems, including polymeric carriers, are being developed to enhance the stability and targeted delivery of these compounds [139].

Emerging evidence also suggests that *Brassicaceae*-derived phytocomplexes may influence gut microbiota and metabolic pathways relevant to glycemic regulation, including modulation of microbial genes such as BT2160 [120]. In parallel, nanotechnology approaches have been explored to improve the bioavailability of poorly absorbed compounds, including flavonoids such as kaempferol, enabling more efficient targeted therapies, particularly in oncology [120,134,157].

Overall, the wide range of culinary, nutraceutical, and pharmaceutical applications of *B. rapa* subsp. *chinensis* reflects its chemical diversity and biological activity. These properties make the species a valuable candidate for the development of functional foods and innovative therapeutic strategies.

### 9.2. Effects of Cooking and Processing on Bioactive Compounds

Post-harvest handling, storage conditions, and culinary processing have a critical impact on the chemical composition and biological activity of *B. rapa* subsp. *chinensis* (Table 15.). These factors influence both the stability of bioactive compounds and their bioavailability, thereby determining the overall nutritional and functional value of the plant [190].

Storage conditions play a key role in preserving plant quality. At room temperature (25 °C), rapid water loss leads to decreased cell turgor and accelerated degradation of secondary metabolites, particularly chlorophylls. In contrast, refrigeration at 4 °C effectively preserves green pigmentation and slows metabolic degradation processes [24]. During storage, accumulation of volatile compounds may occur, altering the sensory profile of the vegetables [34].

Mechanical processing, including cutting and chopping, initiates enzymatic reactions by disrupting cellular compartments. This leads to activation of myrosinase and a reduction in glucosinolate content (18.1–59.2%), accompanied by increased formation of hydrolysis products such as isothiocyanates and nitriles [68,191]. The activity of myrosinase is temperature-dependent, increasing up to approximately 30 °C and then rapidly decreasing due to thermal inactivation [192].

Thermal processing methods differentially affect phytochemical composition. Among them, steaming is considered one of the most favorable techniques, as it preserves a significant proportion of bioactive compounds. Short-term steaming (≤5 min) has been shown to increase the content of phenolic acids, flavonoids (ca. 22%), glucosinolates (ca. 27%), and isothiocyanates (ca. 42%) compared to fresh material, while only partially reducing myrosinase activity (approximately 60–65%) [193].

Blanching, commonly used in industrial processing, partially inactivates myrosinase and reduces glucosinolate content, while maintaining chlorophyll stability under specific conditions, such as the addition of acidic agents (e.g., lemon juice at 95 °C). This process may also increase polyphenol availability [190,194].

In contrast, boiling results in substantial losses of bioactive compounds. High temperatures and direct contact with water lead to complete inactivation of myrosinase, leaching of glucosinolates (20–40%), and significant reductions in isothiocyanates, flavonoids, carotenoids, and vitamins, particularly vitamin C and B-group vitamins [63,152,191].

Frying and high-temperature processing (≈180 °C) also lead to marked degradation of bioactive compounds. Flavonoid content may decrease by approximately 75%, chlorophyll by approximately 70%, and carotenoids by approximately 30%, while vitamin C losses reach approximately 54.9%. Vitamin K content and its bioavailability are also reduced under these conditions [11,48].

Baking and oven processing cause significant reductions in polyphenols (16–62%) and glucosinolates, while promoting structural modifications such as isomerization of flavonoid glycosides [19,195].

Microwave treatment exhibits a dual effect depending on power and exposure time. Low-power, short-term exposure (≤640 W, 15 s) may enhance myrosinase activity and increase isothiocyanate formation, whereas high-power treatment (800 W) leads to enzyme degradation and inhibition of glucosinolate conversion [159].

Freezing represents an effective preservation method, maintaining most glucosinolate levels with minimal changes. In some cases, slight increases in compounds such as neoglucobrassicin and 4-methoxyglucobrassicin have been observed after low-temperature exposure [194,195].

Fermentation induces extensive biochemical transformations mediated by microbial activity, resulting in depletion of glucosinolates and increased formation of isothiocyanates and, to a lesser extent, nitriles. This process also leads to the formation of ascorbigen complexes and modification of polyphenol content, while enhancing certain bioactive properties [153,192,196].

Importantly, although some processing methods reduce the concentration of bioactive compounds, they may simultaneously increase their bioavailability by disrupting plant cell structures and facilitating absorption [11,159].

Overall, the choice of culinary technique significantly determines the final chemical composition of *B. rapa* subsp. *chinensis*. Understanding these transformations enables targeted selection of preparation methods to optimize the retention or formation of specific bioactive compounds, thereby enhancing the functional value of the diet [13].

**Table 15:** Stability of bioactive compounds in *B. rapa* subsp. *chinensis* depending on culinary treatment.

Treatment (or Culinary Process)	Modification/Impact	References
storage	MYROSINASE ↔ until turgor is lost	[108]
storage	GLUCOSINOLATES ↓ ISOTHIOCYANATES ↑	[80]
	CHLOROPHYLL ↓ T = 25 °C; ↔ T = 4 °C	[24]
cutting	MYROSINASE ↓↓↓ GLUCOSINOLATES ↓↓ ISOTHIOCYANATES ↑↑ NITRILLES ↑	[68]
steaming	MYROSINASE ↓↓ t = 5 min. 60–65% GLUCOSINOLATES ↑ t = 5 min.; ↔ t = 30 min. ISOTHIOCYANATES ↑ t = 5 min.; ↑ t = 30 min. TOTAL PHENOLIC ACIDS ↑ t = 5 min.; ↓ t = 30 min. FLAVONOIDES ↑ t = 5 min.; ↓ t = 30 min. VITAMINS ↓ vit. C	[193]
	MINERALS ↓ ~10%	[197]
blanching	MYROSINASE ↓ partly GLUCOSINOLATES ↓↓	[194]
	ISOTHIOCYANATES ↓	[190]
	TOTAL PHENOLIC ACIDS ↑	[194]
	CHLOROPHYLL ↔ with 5% lemon juice; T = 95 °C	[190]
boiling	MYROSINASE ↓↓↓	[152]
	GLUCOSINOLATES ↓↓	[63]
boiling	ISOTHIOCYANATES ↓↓ FLAVONOIDES ↓↓ CHLOROPHYLL ↓↓ CAROTENOIDS ↓	[191]
	VITAMINS ↓↓ vit. C, B9, B1; ↓ vit. B3, A, K	[11,197]
	MINERALS ↓↓	[197]
frying	MYROSINASE ↓↓ GLUCOSINOLATES ↓ FLAVONOIDES ↓↓↓ CHLOROPHYLL ↓↓ CAROTENOIDS ↓	[11]
	VIT ↓↓ vit. C 54,9% ↓ vit. K	[11,48]
baking	MYROSINASE ↓↓↓	[191]
	GLUCOSINOLATES ↓↓	[195]
	EPIOTHIONITRILLES ↑ NITRILLES ↑ TOTAL POLYPHENOLS ↓↓ CHLOROPHYLL ↓↓↓ CAROTENOIDS ↓↓	[19]
microwave	MYROSINASE ↑ 160–640 W, t = 15 s; ↓ 800 W GLUCOSINOLATES ↓ ISOTHIOCYANATES ↑↑ MINERALS ↓	[159]
freezing	MYROSINASE ↓↓	[195]
	GLUCOSINOLATES ↔; ↑ NGBS: from 1.93 ± 0.03 μmol⋅100 g^−1^ FW to 2.39 ± 0,17 μmol⋅100 g^−1^ FW; ↑ 4MGBS from 3.39 ± 0.09 μmol⋅100 g^−1^ FW to 3.80 ± 0.11 μmol⋅100 g^−1^ FW	[194,195]
fermentation	MYROSINASE ↑↑ GLUCOSINOLATES ↓↓↓	[68,196]
fermentation	ISOTHIOCYANATES ↑↑	[192]
	NITRILLES ↑ TOTAL POLYPHENOLS ↓	[196]
	VITAMINS ↔ vit C. in ascorbinogen form	[153,196]

↑—increase, ↓—decrease, ↔—relative stability; scale of intensity: ↑/↓—low, ↑↑/↓↓—medium, ↓↓↓—strong, NGBS—neoglucobrassicin, 4MGBS—4-methoxyglucobrassicin.

### 9.3. Relevance in Supportive Therapy and Skin Health

Improvement of gastrointestinal function, including regeneration of the mucosal barrier and normalization of intestinal transit, is closely associated with systemic health and external physiological outcomes, particularly skin condition. This relationship reflects the importance of the gut–skin axis, whereby dietary patterns influence not only digestive health but also dermatological status [130].

Diets rich in green plant material, including *B. rapa* subsp. *chinensis*, have been associated with visible improvements in skin condition, mediated through enhanced antioxidant status, reduced inflammation, and modulation of gut microbiota [3,49,94]. Maintenance of a balanced intestinal microbiome further contributes to improved neurophysiological function, supporting the broader gut–brain–skin axis. Sulforaphane, a key glucosinolate-derived compound, has been proposed as an important mediator of this interaction, influencing systemic signaling pathways [117].

Traditional practices in rural and indigenous communities have long recognized the therapeutic potential of plant materials, including *Brassicaceae* species, in the treatment of skin disorders such as wounds, eczema, and fungal infections [198]. Contemporary scientific studies support these observations, demonstrating that plant-derived compounds, including flavonoids such as quercetin, promote wound healing and tissue regeneration. Experimental models have shown beneficial effects in conditions such as atopic dermatitis [199].

In addition to flavonoids, glucosinolate-derived compounds also contribute to immunomodulatory and dermatological effects. Administration of 3,3-diindolylmethane (DIM) in experimental models resulted in modulation of immune responses, including regulation of thymus-related hormonal pathways and activation of regulatory T (Treg) cells, supporting immune homeostasis [200]. Sulforaphane has further demonstrated anti-allergic and anti-inflammatory properties, including protective effects on mucosal tissues such as the nasal epithelium [117].

The complex chemical composition of *B. rapa* subsp. *chinensis*, including polyphenols, flavonoids, and sulfur-containing compounds, enables multifaceted biological activity. These compounds may be utilized both internally, through dietary intake, and externally, in the development of topical applications for skin care and therapeutic use [144].

Overall, the relevance of *B. rapa* subsp. *chinensis* in supportive therapy extends beyond basic nutrition, skin health and dermatological applications, immune modulation, and systemic physiological regulation. These properties highlight its potential as a functional food and a source of bioactive compounds for integrative therapeutic strategies.

### 9.4. Use of Pak Choi Extracts in Cosmetics

The growing demand for natural and biocompatible ingredients has led to increasing interest in plant-derived compounds for cosmetic applications. Bioactive substances with antioxidant, anti-aging, anti-inflammatory, and skin-regenerating properties are particularly valued in modern formulations [201]. In this context, plant extracts capable of neutralizing free radicals, improving skin hydration, and enhancing microcirculation are increasingly incorporated into topical products. Chlorophyll represents one of the best-known examples of plant-derived cosmetic ingredients. In addition to its function as a natural colorant, it exhibits deodorizing properties by neutralizing odor-causing compounds and is therefore used in products such as deodorants, oral hygiene solutions, and toothpastes [94,202]. Furthermore, chlorophyll contributes to antioxidant protection, moisture retention, and skin-brightening effects, making it a valuable component of lotions, toners, powders, and daily skincare formulations [203].

The increasing popularity of organic cosmetics reflects consumer concerns regarding exposure to synthetic and potentially harmful ingredients. This trend is particularly relevant among sensitive groups, such as pregnant and breastfeeding women, due to the ability of the skin to absorb and transport chemical substances into the body [129,204]. Consequently, plant-derived ingredients are being explored as safer alternatives for maintaining skin homeostasis and microbiological balance.

Although *B. rapa* subsp. *chinensis* is primarily known for its culinary applications, its potential use in cosmetic formulations is increasingly recognized (Table 16). Various plant parts, including leaves, roots, and seeds, may serve as raw materials for extracts, powders, oils, or fermented products with distinct functional properties. These include antioxidant, soothing, regenerating, anti-allergic, and moisturizing effects, as well as prebiotic activity beneficial for the skin microbiome.

Experimental studies have demonstrated the feasibility of incorporating pak choi extracts into cosmetic formulations. A cream containing ethanolic leaf extract exhibited high stability and safety in both animal and human models, with no observed irritation or intolerance. The presence of flavonoids and glucosinolate derivatives contributed to antimicrobial activity, as well as desirable sensory properties such as uniform consistency and a mild, plant-based fragrance [205].

Regulatory databases further confirm the emerging relevance of *B. rapa* subsp. *chinensis* in cosmetics. Entries in the European CosIng database include fermented extracts (e.g., with *Saccharomyces* or *Lactobacillus*) used as humectants and emollients, as well as powdered leaf forms with antioxidant properties [206,207,208].

Oil derived from pak choi seeds, containing approximately 23.1% lipids with a high proportion of unsaturated fatty acids (including linoleic and oleic acids), also represents a promising ingredient for cosmetic applications. Despite its limited suitability for dietary use due to erucic acid content, this oil exhibits beneficial properties for skin conditioning, including prevention of transepidermal water loss and support of skin regeneration [209,210].

Comparative studies on other *Brassicaceae* species support the potential of pak choi-based formulations. For example, broccoli-derived extracts have been shown to enhance epidermal keratinization and improve skin barrier function [117]. In addition, plant-based cosmetic ingredients are characterized by high biodegradability and environmental compatibility, aligning with sustainability trends in the cosmetics industry [211].

An emerging approach involves the utilization of plant residues and by-products from food processing as sources of cosmetic ingredients. This strategy not only reduces waste but also enables the recovery of valuable bioactive compounds, supporting the development of sustainable and circular production systems [212].

Overall, although the use of *B. rapa* subsp. *chinensis* in cosmetics is still relatively limited, its rich phytochemical composition and diverse biological activities highlight its strong potential for future applications in dermatological and cosmetic formulations.

**Table 16:** Potentiality and directions of cosmetic use of derived raw materials *B. rapa* subsp. *chinensis*.

Plant Raw Material	Cosmetic Form	Direction of Cosmetic Activity	References
Leaves *Brassica rapa chinensis* leaf	extract	soothing antioxidative	[206,213]
	powder	regenerating anti-allergic	[199,207]
	bioferment	nourishing prebiotic	[206,208,213]
	cataplasm	demulcent	[214]
Hairy roots Leaf inoculation with *Agrobacterium rhizogenes*	extract	whitening	[215]
Seeds *Brassica rapa chinensis* seeds	extract	detoxification revitalization	[213,216]
	powder	skin exfoliation	[216]
	oil	anti-aging nourishing regenerating	[209,210]
	cataplasm	anti-swelling	[198]

## 10. Plant Biotechnology Solutions

The scientific literature on in vitro biotechnology of *B. rapa* subsp. *chinensis* reveals a clear imbalance in research focus. Microspore culture systems have been extensively investigated, whereas studies addressing genetic transformation and the enhancement of secondary metabolite production remain relatively limited (Table 17). This disparity may be associated with the inherent recalcitrance of this species to efficient tissue regeneration [217].

Several studies have significantly contributed to the optimization of microspore embryogenesis in this species. For instance, Zhang et al. investigated the effects of histone deacetylase inhibitors on isolated microspore culture (IMC). The supplementation of NLN-13 medium with sodium butyrate (NaB), suberoylanilide hydroxamic acid (SAHA), and trichostatin A (TSA) enhanced embryogenesis and facilitated callus-free plant regeneration. The highest regeneration efficiency was observed in genotype 424 following the addition of 0.05 μM TSA. Similarly, embryos cultured on NLN-13 medium supplemented with 0.05 μM and 0.10 μM SAHA exhibited regeneration rates of 75.01% and 87.30%, respectively. The highest embryo induction was achieved with 2 μM NaB [218].

The importance of genotype-dependent responses in microspore culture has been emphasized by Shumilina et al., who demonstrated that optimal embryo formation was achieved using microspores isolated from flower buds measuring 2.0–2.9 mm in length. Cultivation in liquid NLN medium supplemented with 13% sucrose (w/v), 1% activated carbon, and 24-epibrassinolide significantly improved embryogenesis. Secondary embryo formation was further enhanced on half-strength Murashige and Skoog (MS) medium supplemented with 2% sucrose, 3 g/L Phytagel, and 0.1 mg/L 6-benzylaminopurine (6-BA), resulting in high yields of doubled haploid plants [219].

Earlier work by Cao et al. confirmed that microspore embryogenesis in pak choi is strongly influenced by temperature treatment. A short heat shock at 33 °C for 24 h was found to be essential for embryogenic induction. Under these conditions, embryo yields reached up to 57.4 embryos per bud, and flow cytometric analysis confirmed the formation of haploid plants in selected cultivars [220].

Physical factors such as culture agitation have also been shown to influence embryogenesis. Yang et al. demonstrated that a shaking frequency of 40–50 rpm significantly enhanced the development of cotyledonary embryos and increased overall embryo production by up to 69.37% compared with static cultures. Additionally, shaking reduced culture time by 1–4 days and promoted embryo morphogenesis [221].

Further improvements in microspore culture efficiency have been reported by Gu et al., who demonstrated that modifications of sucrose concentration and medium replacement after 48 h enhanced direct plant regeneration and eliminated the need for subculturing. More than 70% of regenerated plants were spontaneous diploids, and the occurrence of tetraploid plants was also observed [222].

Genotype-dependent variability remains a key factor influencing embryogenesis efficiency. Fang et al. reported significant differences among genotypes and demonstrated that cultivation in liquid NLN-13 medium under rotary shaking at 50 rpm and 25 °C accelerated embryogenesis and increased the proportion of cotyledonary embryos. More than 50% of regenerated plants were confirmed as doubled haploids [223].

The role of growth regulators has also been investigated. Li et al. demonstrated that the application of N-(2-chloro-4-pyridyl)-N′-phenylurea (CPPU) at 0.01 mg/L significantly enhanced microspore embryogenesis, increasing embryo yield by 2.43- to 5.54-fold compared with control conditions [224]. Similarly, thidiazuron (TDZ) supplementation in NLN-13 medium promoted embryogenesis and improved regeneration efficiency, resulting in survival rates of up to 100% and the production of up to 14.67 embryos per bud. This approach enabled the generation of 552 diploid plants and represents a promising strategy for hybrid breeding [225].

In contrast to the extensive research on microspore culture, studies on genetic transformation remain limited. Xu et al. demonstrated Agrobacterium-mediated transformation using *Agrobacterium tumefaciens* strain C58C1 carrying the pBBBast-gus-intron vector. Transformation efficiency was higher in reproductive tissues, particularly flowers, compared with vegetative organs [226].

More recently, genome editing approaches have been applied. Kim et al. successfully employed the CRISPR/Cas9 system targeting BcCENH3 and BcpPLAIIγ genes via Agrobacterium-mediated transformation. Mutation frequencies ranged from 1.7% to 2.1% for BcCENH3 and from 24.5% to 33.7% for BcpPLAIIγ, demonstrating the potential of genome editing strategies in pak choi improvement [227].

In addition to developmental and genetic studies, physiological aspects of in vitro and post-harvest metabolism have also been explored. Song et al. investigated the effects of gibberellic acid (GA) and 6-benzylaminopurine (6-BA) on leaf yellowing and metabolic regulation. These treatments stabilized chlorophyll content and color parameters, enhanced sucrose metabolism, and suppressed pathways associated with protein degradation, autophagy, and chlorophyll catabolism [228].

Overall, current research on *B. rapa* subsp. *chinensis* biotechnology is dominated by studies on microspore embryogenesis and doubled haploid production. In contrast, genetic transformation approaches and strategies aimed at enhancing secondary metabolite production remain underexplored, indicating important directions for future research.

**Table 17:** Key factors influencing microspore embryogenesis and plant regeneration in *Brassica rapa* subsp. *chinensis*.

Factor Category	Experimental Factor	Conditions Applied	Observed Effect	Reference
Temperature (stress induction)	Heat shock	33 °C (24 h)	Induction of embryogenesis; up to 57.4 embryos per bud; haploid formation confirmed	[220]
Epigenetic modifiers	TSA, SAHA, NaB	0.05–2 μM	Increased embryo yield and regeneration; up to 87.30% regeneration efficiency	[218]
Growth regulators	CPPU	0.01 mg/L	Increased embryogenesis (2.43–5.54-fold)	[224]
	TDZ	various concentrations	Enhanced embryogenesis; up to 14.67 embryos per bud; high regeneration efficiency (81.67%)	[225]
Medium composition	NLN medium + 13% sucrose	liquidculture	Improved embryogenesis and embryo development	[219]
	½ MS medium + 6-BA	2% sucrose, 0.1 mg/L 6-BA	Increased secondary embryo formation	[219]
Physical conditions	Shaking culture	40–50 rpm	Increased embryo production (up to 69.37%); accelerated development	[221]
Carbon source optimization	Sucrose adjustment	reduced after 48 h	Enhanced direct plant regeneration; reduced need for subculture	[222]
Genotype effect	Different cultivars/lines	various genotypes	Strong variability in embryogenesis and doubled haploid production (>50%)	[223]
Polyploid formation	IMC-derived plants	Spontaneous processes	High frequency of spontaneous diploids and occurrence of tetraploids	[222]
Genetic transformation	*Agrobacterium tumefaciens*	Vector pBBBast-gus-intron	Higher transformation efficiency in reproductive tissues	[226]
Genome editing	CRISPR/Cas9	Targeting BcCENH3, BcpPLAIIγ	Mutation frequency: 1.7–33.7% depending on gene	[227]

## 11. Limitations and Perspectives

Key limitations include the lack of standardisation in genetic factors (particularly seed origin), as well as climatic, agronomic, and cultivation practices. An additional important consideration is the inherent variability of the plants themselves, which results in highly heterogeneous chemical profiles among cultivars of the same species. While a substantial body of experimental data has been generated under controlled growing conditions, evidence concerning long-term human applications remains scarce. Nevertheless, these constraints open a broader avenue for future investigations aimed at elucidating aspects that have not yet been adequately characterised.

## 12. Conclusions

*B. rapa* subsp. *chinensis* is a nutritionally and phytochemically diverse plant characterized by a complex profile of glucosinolates and their hydrolysis products, phenolic compounds, carotenoids, vitamins, minerals, and other primary and specialized metabolites. The available evidence indicates that its functional potential cannot be attributed to a single compound or phytochemical class but rather reflects the combined contribution of multiple constituents. Importantly, their qualitative and quantitative profiles are highly dependent on genotype, developmental stage, cultivation conditions, environmental factors, and post-harvest processing, highlighting compositional variability as a key consideration when interpreting the biological properties of pak choi.

Current evidence supports antioxidant, anti-inflammatory, metabolic-regulatory, microbiota-modulating, and chemopreventive potential associated with pak choi and its characteristic constituents. However, the strength of evidence differs considerably among these effects, and findings obtained for isolated phytochemicals or other *Brassicaceae* species should not be directly extrapolated to pak choi. In particular, the limited availability of pak choi-specific human studies, together with insufficient knowledge of bioavailability, metabolism, dose–response relationships, and long-term effects, currently restricts translation of experimental findings into evidence-based nutritional or pharmacological recommendations.

A major future challenge is therefore to establish robust relationships between phytochemical composition and biological activity through the integration of metabolomics, transcriptomics, functional assays, and appropriately designed in vivo and clinical studies. Greater standardization of plant material and experimental conditions will also be essential to account for genotype- and environment-dependent variability. At the same time, improved understanding of processing-induced transformations, gut microbiota-mediated metabolism, and compound bioavailability will be critical for defining how the phytochemical potential of pak choi translates into biological effects after consumption.

Beyond its established dietary importance, pak choi offers promising opportunities for the development of functional foods, nutraceuticals, and cosmetic ingredients, while plant biotechnology provides additional possibilities for crop improvement and the targeted enhancement of valuable phytochemical traits. Integration of conventional cultivation with in vitro approaches, elicitation, metabolic engineering, and genome-editing technologies may further expand the potential of this species. Taken together, the evidence positions pak choi not simply as a nutrient-dense leafy vegetable, but as a multifunctional plant resource whose future value will depend on bridging phytochemical diversity with reproducible biological evidence and biotechnology-driven innovation.

## Figures and Tables

**Table 1:** Genetics of selected plants from Brassicaceae family.

Species Common Name	Subspecies—Examples	Ploidy	Genome
*Brassica nigra* (L.) Koch Black mustard	var. *nigra* var. *abyssinica* var. *bracteolata*	2*n* = 8 or 2*n* = 16	B or BB
*Brassica oleracea* L. Cabbage	var. *oleracea* var. *capitata* var. *italica* var. *sabellica*	2*n* = 9 or 2*n* = 18	C or CC
*Brassica rapa* L. (in the past *Brassica campestris*) Turnip rape	var. *rapa* var. *oleifera* var. *pekinensis* var. *chinensis*	2*n* = 10 or 2*n* = 20	A or AA

**Table 2:** Examples of Chinese cultivated varieties of *Brassica rapa* var. *chinensis*.

Morphological Appearance	Varieties
Light purple leaves	Jinzuanziyi
Purple leaves	Zijinkuaicai, Zijinyoucai, Zijiaren, Ziyuyoucai
Light green leaves	Dongyue, Shanghaiqing
Dark green leaves	Heiyoubaicai, Heimeigui, Kuishanheiyetianbaicai
Emerald petiole	Feicuiyihao
Green petiole	Jingpinwuyueman, Xinguanhua, Yuexiaqinggengcai, Jingguanerhao, Dongchunsansan
White petiole	Shuoyuannaibaicai, Sucuibai, Heiniu, Lifei, Zhongyannaibaicai
White-green petiole	Qingshuangjimaocai
Hypertrophic petiole	Meihuiqinggengbaicai, Feicuierhao, Xiazhijiao, Guoxiayihao
Long petiole	Naiyouxiaobaicai
Short petiole	Woyouganju, Huangxinwu, Heixinwu, Aijiao, Teaibaixue
Narrow petiole	Lexialiangpin
Slender petiole	Ruiguanqingbang, Lvsongxiaobaicai, Baigengsijixiaobaicai

**Table 3:** Cultivated varieties of *Brassica rapa* subsp. *chinensis* represented in the collection of the N.I. Vavilov All-Russian Institute of Plant Genetic Resources (VIR).

Varieties	Morphological Appearance
Syusman	compact leaf rosette, a thick, relatively short and very wide, light green leaf petiole
Taisai	closed leaf rosette with leaves and a long, pale green or white leaf petiole
Piorbai	semi-spreading leaf rosette with numerous, dark green leaves with a medium-long, light green petiole
Leychoi	shiny, dark green, convex leaves with a thick, white petiole
Joychoi	small, spreading or semi-spreading leaf rosette with slightly upright, very compact, fragile leaves

**Table 4:** The content of nutrients and water reported in *B. rapa* subsp. *chinensis*.

Nutrient	Contents in Pak Choi (per 100 g Fresh Weight)				High-Yielding Varieties —Examples (with Origin) [7,16,46,54]	Daily Requirement [55,56,57]	Potential Health Effect [1,20,55,56,58]
	Mature Plants		Microgreens				
	[6,7,11,59]	[46,60]	[61]	[54,62]			
water	76.0–92.5 g	96.1–96.8 g	93.7 g	94.4 g	‘Pak Choi White’ (Vietnam), ‘Dwarf Pakchoi’, ‘Thailand Fast’ (China), ‘Xia Hua’ (Hong Kong)	does not apply	Hydration, support of detoxification processes in the liver
fiber	2.7 g	1.0 g	10.8 g DW	-	especially in many microgreens; ‘Black Shiny Winter’ (China), ‘Taisai’ (Japan)	about 30 g/day	Regulation of intestinal function, support of maintaining a normal body weight, support of maintaining stable blood glucose levels
carbohydrates	3.1 g	2.2 g (include 0.8–1.2 g sugars)	-	-	-	individual	Providing energy substrates to the body
protein	1.7 g	1.5 g	2.3 g DW	1.9–3.0 g	‘Xiangqingcai’, ‘Naibaicai’ (China)	0.66 g/kg/day–0.83 g/kg/day for adults 1.0–1.5 g/kg/day for older adults (≥65 years)	Nutrition, regulation of hunger, support of skin appearance
fat	0.2 g	0.2 g	3.4 g DW	-	-	individual	Energy supply, facilitation of vitamin absorption
total glucosinolates	740–3820 µmol DW	229.0 µmol DW	3818 µmol	257 µmol	‘Shanghai Late May’, ‘Shanghai Late April’ (China)	does not apply	Better metabolism and beneficial gut flora, health skin
total phenolic compounds	470.2–881.7 µmol GAE	99.9 µmol DW green cultivars 323.3 µmol DW purple cultivars	131.4 mg GAE	145.4 mg GAE	‘Red Wizard F1’ (United Kingdom), ‘Qianyecai’, ‘Maertou’ (China)	does not apply	Brighter skin, neutralizing free radicals, more stable blood glucose levels, and proper lipid metabolism
ascorbic acid (vitamin C)	53.0 mg	45.0 mg	189.5 mg DW	28.9 mg	‘Green Fortune’ (Japan), ‘Xiangqingcai’, ‘Bottle Long Petioled White’ (China), ‘Gracious Pai-Tsai’ (Taiwan)	60 mg women/day 75 mg men/day	Antioxidation, support of metabolic processes
calcium	102.0 mg	105.0 mg	-	58 mg	‘Dwarf Erect Pakchoi’, ‘Golden Early No. 1 Sweet Pakchoi’, ‘Zhu Pob Tsai’ (China)	800 mg/day	Strengthening, mineralization of bones
provitamin A (beta-caroten form)	2.3 mg	2.7 mg	0.4 mg	5.1 mg	‘Dwarf Black Behay’, ‘Green Fortune’, ‘Wu Ta Tsai’, ‘Maertou’ (China)	500–625 μg RE women/men	Proper eye function, support of regeneration processes (e.g., skin, respiratory tract lining), impact on fertility

**Table 5:** Diversity and quantitative variability of polyphenolic compounds identified in *B. rapa* subsp. *chinensis*, including representative cultivars and concentration ranges.

Compound	Chemical Formula	GAE Content [µg/mL]	Varietes	References
5-*p*-coumaroylquinic acid	C_16_H_18_O_8_	4.77–9.41	Nd	[59]
caffeic acid	C_9_H_8_O_4_	0.7–9.23	‘Qianyecai’	[16,59]
caffeoylmalic acid	C_13_H_12_O_8_	8.81–13.96	Nd	[59]
caffeoyltartaric acid	C_13_H_12_O_9_	0.58–1.28	Nd	[59]
chlorogenic acid	C_16_H_18_O_9_	3.45–6.45	‘Qianyecai’	[16,59]
cinnamic acid	C_9_H_8_O_2_	nd	‘Paopaoqing’, ‘Naibaicai’	[16]
coumaric acid	C_9_H_8_O_3_	0.18–0.34	Nd	[59]
ferulic acid	C_10_H_10_O_4_	0.48–0.8	‘Qianyecai’	[16,59]
homovanilloyquinic acid	C_9_H_10_O_4_	nd	‘Xiangqingcai’, ‘Paopaoqing’	[16]
isorhamnetin-3-gentiobioside-7-glucoside	C_34_H_42_O_22_	0.08–0.43	‘Qianyecai’	[16,59]
phillyrin	C_27_H_34_O_11_	0.12–0.26	Nd	[59]
protocatechuic acid	C_7_H_6_O_4_	0.49–0.9	‘Maertou’	[16]
rosmarinic acid	C_18_H_16_O_8_	nd	‘Qianyecai’	[16]
sinapic acid	C_11_H_12_O_5_	0.49–2.49 mg∙g^−1^ DM	pak choi #16, ‘Qianyecai’	[9,16]

nd—no data.

**Table 6:** Major flavonoid compounds identified in *B. rapa* subsp. *chinensis*.

Compound	Chemical Formula	References
delphinidin 3-O-glucoside	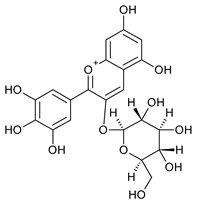	[77]
isorhamnetin	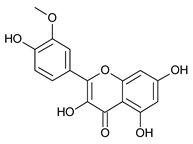	[9,16,76,78]
isorhamnetin-3,7-di-O-glucoside	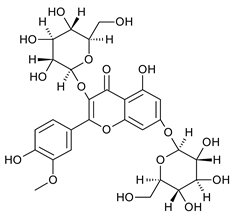	[76]
isorhamnetin-3-O-glucoside	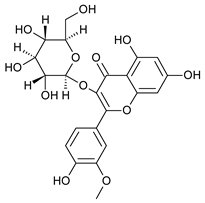	[76]
kaempferol	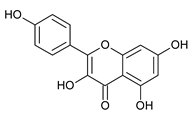	[9,16,76,78]
kaempferol 3-O-caffeoyl-7-O-glucoside	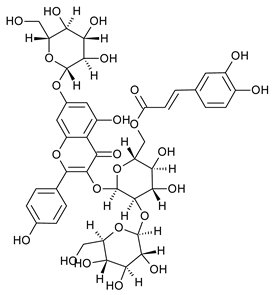	[76]
kaempferol 3-O-feruloyl-sophoroside 7-O-glucoside	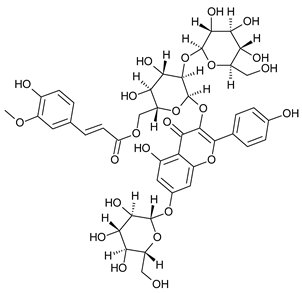	[76]
kaempferol 3-O-hydroxyferuloyl-glucoside-7-O-glucoside	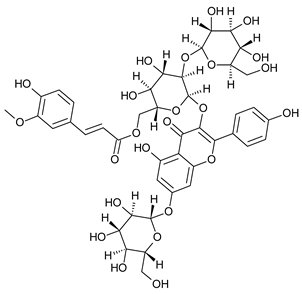	[76]
kaempferol 3-O-sinapoyl-sulphate-7-O-glucoside	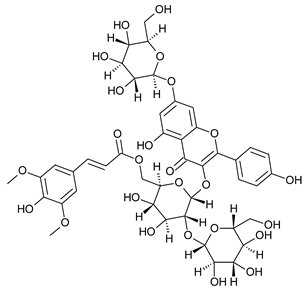	[76]
kaempferol-3,7-di-O-glucoside	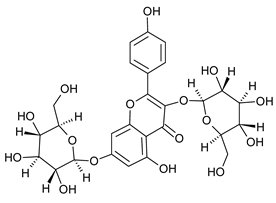	[76]
quercetin	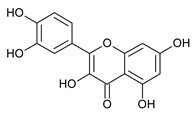	[9,16,76,78]

**Table 7:** Glucosinolates identified in *B. rapa* subsp. *chinensis*.

Class	Name (Abbreviation)	Chemical Formula	Average Content	Raw Material	References
AROMATIC	Glucotropaeolin (GTL)	C_14_H_19_NO_9_S_2_	6.451 μmol∙kg^−1^ DW	leaf	[34]
	Gluconasturtiin (GNT)	C_15_H_21_NO_9_S_2_	678.72 μmol∙kg^−1^ DW	leaf	[34]
	Glucobarbarin (GBB/BAR)	C_15_H_21_NO_10_S_2_	3.171 μmol⋅kg^−1^ DW	leaf	[34]
	Sinalbin (SB)	C_30_H_42_N_2_O_15_S_2_	0.086 μmol⋅kg^−1^ DW	leaf	[34]
ALIPHATIC	Glucoiberin (GIB)	C_11_H_21_NO_10_S_3_	0.17 μmol⋅g^−1^ DW	leaf	[13]
			0.375 μmol⋅kg^−1^ DW	leaf	[34]
	Sinigrin (SIN/SG)	C_10_H_17_NO_9_S_2_	4.722 μmol⋅kg^−1^ DW	leaf	[34]
	Glucocheirolin -	C_11_H_21_NO_11_S_3_	5.256 μmol⋅kg^−1^ DW	leaf	[34]
	Glucoerucin (GER)	C_12_H_29_NO_9_S_3_	49.366 μmol⋅kg^−1^ DW	leaf	[34]
	Glucoraphanin (GRA/GRP)	C_12_H_23_NO_10_S_3_	172.591 μmol⋅kg^−1^ DW	leaf	[34]
	Gluconapoleiferin (GNLa/GNL)	C_12_H_21_NO_10_S_2_	0.73 mg∙g^−1^ DW	sprout	[86]
	Gluconapin (GNA)	C_11_H_19_NO_9_S_2_	0.37 μmol⋅g^−1^ DW	leaf	[13]
			4.5 mg⋅g^−1^ DW	sprout	[86]
	Progoitrin (PRO)	C_11_H_19_NO_10_S_2_	17.84 mg⋅g^−1^ DW	sprout	[86]
			1132.364 μmol⋅kg^−1^ DW	leaf	[34]
	Epiprogoitrin (EPI)	C_11_H_19_NO_10_S_2_	843.059 μmol⋅kg^−1^ DW	leaf	[34]
	Glucoraphasatin (GRH)	C_12_H_21_NO_9_S_3_	0.231 μmol⋅kg^−1^ DW	leaf	[34]
	Glucoraphenin (GRE)	C_12_H_21_NO_10_S_3_	0.981 mg∙g^−1^ DW	leaf	[34]
	Glucoberteroin (GBT/GBE)	C_13_H_25_NO_9_S_3_	148.188 μmol⋅kg^−1^ DW	leaf	[34]
	Glucobrassicanapin (GBN)	C_12_H_21_NO_9_S_3_	1.46 μmol⋅g^−1^ DW	leaf	[13]
			3139.729 μmol⋅kg^−1^ DW	leaf	[34]
			1.96 mg⋅g^−1^ DW	sprout	[86]
INDOLIC	Glucobrassicin (GBS/GLB)	C_16_H_20_N_2_O_9_S_2_	0.09 μmol⋅g^−1^ DW	leaf	[13]
			0.6 mg⋅g^−1^ DW	sprout	[86]
	Neoglucobrassicin (NEO/NGB/neoGBS)	C_17_H_22_N_2_O_10_S_2_	0.07 μmol⋅g^−1^ DW	leaf	[13]
			3.22 mg⋅g^−1^ DW	sprout	[86]
	4-Methoxyglucobrassicin (4MGBS)	C_17_H_22_N_2_O_10_S_2_	0.13 μmol⋅g^−1^ DW	leaf	[13]
			2.89 mg⋅g^−1^ DW	sprout	[86]

**Table 8:** Formation of isothiocyanates from glucosinolates via myrosinase-catalyzed hydrolysis in disrupted tissues of *B. rapa* subsp. *chinensis*.

Classification	Glucosinolate name	Isothiocyanate name
	SUBSTRATUM before conversion	PRODUCT after conversion
AROMATIC	Glucotropaeolin	benzyl isothiocyanate
	Gluconasturtiin	phenethylisothiocyanate
	Sinalbin	4-hydroxybenzyl isothiocyanate
ALIPHATIC	Sinigrin	allylisothiocyanate
	Glucocheirolin	thioaliphatic nitrile
	Glucoerucin	Erucin
	Glucoraphanin	sulforaphane
	Gluconapin	3-butenyl isothiocyanate
	Glucobrassicanapin	4-pentenyl isothiocyanate
	Progoitrin	Goitrin
INDOLIC	Glucobrassicin	indole-3-carbinol
	Neoglucobrassicin	n-methoxyindolyl-3-carbinol
	4-Methoxyglucobrassicin	nd *

* nd—no data.

**Table 9:** Content of major plant pigments in *B. rapa* subsp. *chinensis*, determined in different studies and at different developmental stages.

Compounds	Microgreens		Leaves	
	Content	References	Content	References
Chlorophyll *a*	6.13 µg∙g^−1^ FW	[61]	0.32 mg∙g^−1^ FW	[73]
	8217.7 µg∙g^−1^ FW	[19]	338.45–473.22 mg∙100 g^−1^ DW	[59]
			14,586.5 µg∙g^−1^ FW	[19]
Chlorophyll *b*	3.00 µg∙g^−1^ FW	[61]	0.114 mg∙g^−1^ FW	[73]
	1428.3 µg∙g^−1^ FW	[19]	121.31–215.44 mg∙100 g^−1^ DW	[59]
			2043.8 µg∙g^−1^ FW	[19]
Total chlorophyll (*a* + *b*)	15.99 µg∙g^−1^ FW	[61]	459.76–683.69 mg∙100 g^−1^ DW	[59]
	9646 µg∙g^−1^ FW	[19]	16,630.3 µg∙g^−1^ FW	[19]
			0.47–1.45 g∙kg^−1^ FW	[99]
Lutein	2.7 mg∙100 g^−1^ FW	[62]	785.8 µg∙g^−1^ FW	[19]
	519.5 µg∙g^−1^ FW	[19]	~43% total carotenoids	[7]
	27.48–50.35 mg∙100 g^−1^ FW	[93]	200–230 ng∙mg^−1^ DW	[98]
Beta-carotene	5.1 mg∙100 g^−1^ FW	[62]	429.4 µg∙g^−1^ FW	[19]
	194.2 µg∙g^−1^ FW	[19]	173–189 µg∙g^−1^ DW	[7]
	1.85–3.34 mg∙100 g^−1^ FW	[93]		
Neoxanthin	149.9 µg∙g^−1^ FW	[19]	193.7 µg∙g^−1^ FW	[19]
	2.08–20.64 mg∙100 g^−1^ FW	[93]	~13% total carotenoids	[7]
Violaxanthin	0.4 mg∙100 g^−1^ FW	[62]	401 µg∙g^−1^ FW	[19]
	142.1 µg∙g^−1^ FW	[19]	~28% total carotenoids	[7]
	11.10–60.56 mg∙100 g^−1^ FW	[93]		
Total carotenoids	3.74 µg∙g^−1^ FW	[61]	18.45–41.23 mg∙100 g^−1^ DW	[59]
	1005.7 µg∙g^−1^ FW	[19]	0.21 mg∙g^−1^ FW	[73]
	8.2 mg∙100 g^−1^ FW	[62]	440–2020 µg∙g^−1^ DW	[7]
Total anthocyanins	0.1–0.3 mg∙g^−1^ DW	[66]	0–735.39 mg·kg^−1^	[96]
	4.11 µmol∙100 g^−1^ FW	[61]	green variety 0–26% total flavonoids purple variety ~50% total flavonoids	[77]

**Table 10:** Selected proteinogenic amino acids identified in *B. rapa* subsp. *chinensis* and their relative abundance.

Type	Compound	Intensity [Ratio/g]
Proteinogenic amino acids	Alanine (Ala)	6.42 ± 2.94
	Valine (Val)	15.82 ± 6.34
	Isoleucine (Ile)	10.66 ± 4.57
	Leucine (Leu)	7.84 ± 3.76
	Methionine (Met)	2.25 ± 0.18
	Phenylalanine (Phe)	6.06 ± 0.26
	Threonine (Thr)	9.22 ± 1.10
	Tryptophan (Trp)	1.12 ± 0.17

**Table 14:** Selected contraindications and potential dietary limitations associated with consumption of *B. rapa* subsp. *chinensis*.

Problem or Possible Negative Co-Interaction	Case	Solution	References
Hashimoto’s disease	limitation of iodine absorption from food, occurrence of thyroid goiter	reducing the content of goitrogens through appropriate culinary processing	[68,140,179]
Swallowing problems	too hard and large pieces	change in serving consistency	[111,180]
Dysphagia			
Poor oral health	crunchy, hard texture of leaf petiole		
Dental cavities			
Acute gastrointestinal disorders	digestive system hyperresponsiveness	introduction to the diet in small portions only in cooked form	[23,140,149]
Use of anticoagulant medications	antagonistic effects of drugs and vitamin K	maintaining a time gap between taking medications and eating	[140]

## Data Availability

No new data were created or analyzed in this study. Data sharing is not applicable to this article.

## Abbreviations

The following abbreviations are used in this manuscript:

6-BA	6-benzyladenine
ABTS	2,2′-azinobis(3-ethylbenzothiazoline-6-sulfonic acid)
ABA	abscisic acid
AHR	aryl hydrocarbon receptor
CPPU	N-(2-chloro-4-pyridyl)-N′-phenylurea
CRISPR	Clustered Regularly Interspaced Short Palindromic Repeats
DPPH	2,2-diphenyl-1-picrylhydrazyl
FRAP	ferric reducing antioxidant power
GA	gibberellic acid
GSLs	glucosinolates
HPLC	high-performance liquid chromatography
IMC	isolated microspore culture
LC-MS/MS	liquid chromatography tandem mass spectrometry
LED	light-emitting diode
MS medium	Murashige and Skoog medium
NaB	sodium butyrate
NLN medium	Nitsch and Nitsch medium, developed by R. Lichter
ORAC	oxygen radical absorbance capacity
PGRs	plant growth regulators
ROS	reactive oxygen species
SAHA	suberoylanilide hydroxamic acid
SI	sulfur index
TDZ	thidiazuron
TFC	total flavonoid content
TPC	total phenolic content
TSA	trichostatin A
UHPLC	ultra-high-performance liquid chromatography
